# Cu_2_PdSnSe_4_ and Cu_2_PdSn(S,Se)_4_ Palladium-Substituted Kesterite Nanomaterials for Thin-Film Solar Cells

**DOI:** 10.1021/acsmaterialsau.4c00129

**Published:** 2025-02-11

**Authors:** Kelechi Nwambaekwe, Sodiq Yussuf, Ziyanda Tshobeni, Chinwe Ikpo, Jaymi January, Meleskow Cox, Precious Ekwere, Shimelis Admassie, Xinwen Peng, Emmanuel Iwuoha

**Affiliations:** † South African Research Chair for NanoElectrochemistry & Sensor Technology, Fourth Floor Chemical Sciences Building, University of the Western Cape, Robert Sobukwe Road, Bellville, 7535 Cape Town, South Africa; ‡ Department of Chemical Sciences, Olabisi Onabanjo University, P. M. B. 2002, Ago-Iwoye, Ogun State 120107, Nigeria; § Department of Chemistry, Addis Ababa University, P.O. Box 1176, Addis Ababa 1176, Ethiopia; ∥ State Key Laboratory of Pulp and Paper Engineering, South China University of Technology, Guangzhou 510640, Guangdong, China

**Keywords:** defect concentrations, kesterite solar cell, polyol microwave synthesis, solar cell, superstrate photovoltaic cell

## Abstract

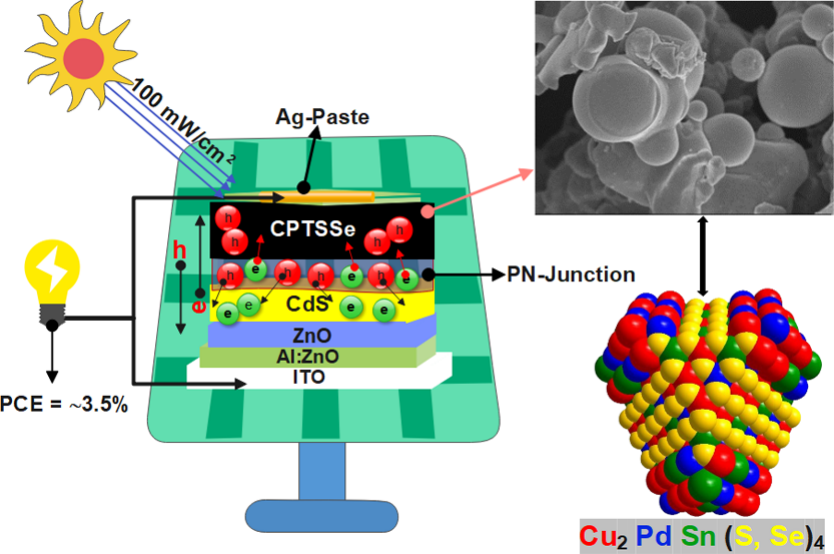

Kesterites are being studied intensively as sustainable absorber materials for solar cell development. However, elements such as Zn and Cu exhibit antisite defects that function as charge traps and recombination centers that affect the light absorption and carrier transport efficiencies of kesterite solar cells. The substitution of Zn or Cu with other metals is one of the strategies used to improve the photovoltaic performance of kesterites. This study focuses on the preparation and photovoltaics of Cu_2_PdSnSe_4_ (CPTSe) and Cu_2_PdSn(S,Se)_4_ (CPTSSe) kesterite nanoparticles (containing Pd instead of Zn) by a modified solvothermal (polyol) microwave synthesis method. The nanomaterials exhibited a tetragonal kesterite crystal structure with polydispersed morphology and average crystallite sizes of 22 and 17 nm for CPTSe and CPTSSe, respectively. DAMMIF ab initio analysis of the small-angle X-ray scattering data determined the shape of CPTSe and CPTSSe nanomaterials to be ellipsoidal. Ultraviolet–visible (UV–vis) spectroscopy revealed red-shift absorption properties, with bandgap energy values of 1.13 eV (CPTSe) and 1.20 eV (CPTSSe), thereby making them suitable light absorber materials for photovoltaic applications. Photoluminescence spectroscopy characterization confirmed the attenuation of defect concentrations in CPTSe and CPTSSe compared to the Zn analogue, which positively impacts the charge-carrier transport and recombination properties. A preliminary test of the materials in superstrate photovoltaic cell devices yielded power conversion efficiency values of 1.32% (CPTSe) and 3.5% (CPTSSe). The CPTSe- and CPTSSe-based photovoltaic devices maintained ∼70% mean open-circuit voltage (*V*oc), which is a significant improvement over the ∼20% *V*_oc_ retained by Zn-based kesterites after 24 days.

## 1 Introduction

The interest in metal chalcogenide thin films such as cadmium telluride (CdTe) and copper indium gallium sulfide (CIGS) in solar cell engineering lies in their remarkable versatility. Their band gaps can be tailored, enabling optimal light absorption across a wide range of the solar spectrum. This direct tunability coupled with their ability in achieving high energy conversion efficiencies presents them as contenders in the quest for efficient and sustainable photovoltaic (PV) technologies.^1^ Despite commercialization, chalcogenide-based thin films present significant, futuristic challenges. They are hampered by the dependence on rare earth elements such as In and Ga in CIGS, raising concerns about sustainability and supply chain longevity. Additionally, the presence of toxic elements such as Cd in CdTe raises environmental and health concerns. Addressing these limitations, through material substitution and exploration of nontoxic alternatives, is crucial for the long-term viability and widespread adoption of these promising technologies.^2^ These challenges led to the exploration of copper zinc tin sulfide selenide (CZTSSe) (known by the most stable phase, kesterite) for use in thin-film PV cells, as its constituent elements are earth-abundant and nontoxic.^3^ Additional desirable attributes of CZTSSe materials include a high absorption coefficient (>10^4^ cm^–1^) and an optimal direct-tunable band gap of 1.5 eV.^4^ The evolution of kesterite-based solar cells has been remarkable, witnessing a meteoric rise (over a brief period) in the power conversion efficiency (PCE) from 0.6 to 12.6% for pristine CZTSSe absorber layers. This drastic improvement underscores the potential of these materials due to their direct bandgap tunability and favorable optoelectronic properties. However, further optimization beyond the current 12.6% requires a nuanced understanding of the intricate interplay between their stoichiometric compositions, crystal structures, and defect formations. Addressing these challenges through material engineering and targeted defect mitigation strategies holds the key to unlocking the potential of kesterite-based photovoltaics, paving the way for their future as efficient and sustainable solar energy materials.^5,6^ The crystal structure of CZTSSe plays a pivotal role in its effectiveness in PV applications. While three phases exist (kesterite, stannite, and wurtzite), the tetragonal kesterite phase exhibits the desired stability and electronic properties for PV applications.^2^ However, achieving pure kesterite during synthesis presents a significant challenge due to its narrow formation window within the stoichiometric space.^2^ This limitation often leads to the formation of secondary phases such as ZnS/Se, Cu_2_S/Se, and Cu_2_ZnSn_3_S/Se_8_, whose formation depends on specific Zn-rich, Cu-rich, and Sn-rich or -poor conditions.^2,7−9^ These secondary phases, such as Cu-rich induced Cu_2_S/Se, can introduce detrimental effects such as shunt losses through increased conductivity.^2,10^ Further complications include intrinsic crystal defects such as V_Cu_ vacancies and Cu_Zn_ antisite defects, arising from the similar atomic radii of Cu and Zn. V_Cu_ vacancies are responsible for a deep donor level, resulting in a reduced bandgap energy.^11^ These defects function as charge traps and recombination centers, impacting light absorption and carrier transport and hindering device performance. The persistent open-circuit voltage deficit (*V*_oc,def_) in CZTSSe devices is largely attributed to these antisite defects.^2,12^ Extensive research suggests that Cu-poor and Zn-rich synthesis conditions can mitigate defect formation, leading to improved device performance.^10^

Since the discovery of its PV potential, researchers have sought to overcome its inherent performance limitations. Strategies such as elemental doping and alloying have been employed,^13−19^ with partial or complete anion substitution of sulfur with selenium (CZTSSe) achieving an efficiency of 12.6%.^20^ However, progress has stagnated since this milestone, prompting the exploration of the cation substitution approach. This method has recently yielded a remarkable 18.63% efficiency in a device with 30% Ag substitution of Cu atoms.^21^ In this work, a cation substitution strategy was employed via microwave solvothermal synthesis to produce copper palladium tin selenide (CPTSe) and copper palladium tin sulfide selenide (CPTSSe) nanomaterials. Alongside, copper zinc tin selenide (CZTSe) and copper zinc tin sulfide selenide (CZTSSe) counterparts were prepared, enabling a comprehensive investigation of the impact of complete Zn-to-Pd substitution on the optical, electronic, crystallographic, and phase stability characteristics as well as the PV device performance.

## 2 Experimental Procedures

### 2.1 Synthesis of Cu_2_PdSn(S,Se)_4_ and Cu_2_ZnSn(S,Se)_4_ Nanoparticles

In the synthesis of CPTSe, CPTSSe, CZTSe, and CZTSSe nanoparticles, we employed a modified solvothermal (polyol) microwave method.^22^ All precursors were procured from Sigma-Aldrich and used without further purification. A mixture containing 1.68 mmol of CuCl, 1.1 mmol of ZnCl_2_ (for CZTSe and CZTSSe) or PdCl_2_ (for CPTSe and CPTSSe), 1.0 mmol of SnCl_4_·5H_2_O, and 3.78 mmol of Se (for CZTSe and CPTSe) or 3.78 mmol of a 3:1 S:Se mixture (using thiourea as the sulfur source, for CZTSSe and CPTSSe) was prepared in a 30 mL diethylene glycol solution in a glass vial. This mixture was then transferred to a Teflon vessel and subjected to microwave irradiation under controlled power conditions (770 W). The microwave was programmed to ramp up to 770 W within 5 min, followed by a 20 min reaction period with continuous stirring. The reaction was then automatically cooled down for 25 min, bringing it to completion. The resulting nanoparticles were precipitated by dilution with isopropanol and centrifugation at 4000 rpm for 10 min. This washing and centrifugation process was repeated twice, and the final products were dried in an oven at 100 °C for 6 h. The collected nanoparticles could be redispersed in isopropanol for further analysis.

### 2.2 Preparation of Kesterite PV Cell Devices

The kesterite PV devices were developed in accordance with the superstrate architecture. Onto a precleaned indium tin oxide (ITO)-patterned soda-lime glass (SLG) substrate, the window layers were sequentially deposited: a transparent conductive oxide layer of aluminum-doped zinc oxide (Al:ZnO) followed by an intrinsic zinc oxide (*i*-ZnO) layer. This bilayer structure enhances light transmittance and provides electron extraction capabilities. Next, a buffer layer of cadmium sulfide (CdS) was deposited to facilitate efficient charge-carrier transport and passivate the kesterite absorber surface. Finally, the p–n junction was formed by depositing kesterite nanoparticle inks onto the CdS layer. The device fabrication was completed by coating a conductive silver paste cathode, resulting in the following final architecture: ITO/Al:ZnO/*i*-ZnO/CdS/CPTSe (CPTSSe) or CZTSe (CZTSSe)/Ag-paste.

#### 2.2.1 Deposition of the Al:ZnO and *i*-ZnO (n-Type) Window Layer

Regarding the materials and methods used in this study, commercially available Al-doped ZnO (AZO) and intrinsic ZnO (*i*-ZnO) nanoparticles dispersed in isopropanol to form stable nanoinks were purchased from Sigma-Aldrich. The deposition process utilized precleaned ITO-coated SLG substrates, with spin-coating as the chosen technique. Following deposition, heat treatment was performed by using a hot plate in ambient air. For the AZO layer, the dispersion was spin-coated onto the ITO substrate at 1500 rpm for 30 s, resulting in a uniform thin film, and immediately subjected to heat treatment at 200 °C for 2 min to improve adhesion and crystallinity, achieving a final thickness of approximately 150 nm. Subsequently, the *i*-ZnO nanoinks were spin-coated onto the AZO layer using the same method, achieving a thickness of about 50 nm. The resulting bilayer structure of AZO/*i*-ZnO forms the complete window layer of the kesterite PV device, where the AZO layer provides high electrical conductivity and transparency, and the *i*-ZnO layer enhances light transmittance and electron extraction capabilities.

#### 2.2.2 Deposition of the CdS (n-Type) Buffer Layer

The CdS nanopowder used in this study, procured from Sigma-Aldrich, was solubilized in 2-propanol to form a solution for deposition. The substrate preparation involved the deposition and annealing of window layers (AZO/*i*-ZnO) before the CdS buffer layer deposition using the spin-coating technique. The CdS solution was sonicated to ensure effective dispersion of the nanopowder and then spin-coated onto the window layers at 1500 rpm for 30 s to create a thin, uniform film. To enhance film adhesion, crystallinity, and electrical properties, the coated substrate was subjected to thermal annealing at 200 °C for 5 min on a hot plate in ambient air, resulting in a CdS buffer layer that was approximately 60 nm thick. This CdS layer serves as a buffer between the n-type window layers and the p-type kesterite absorber, facilitating efficient transport of photogenerated electrons from the absorber to the n-type layers, thereby enhancing the device performance. Furthermore, it passivates the kesterite surface, reducing surface defects and improving carrier lifetime.

#### 2.2.3 Deposition of the CPTSe, CPTSSe, CZTSe, and CZTSSe (p-Type) Absorber Layer

A 150 mg portion of each kesterite nanomaterial CPTSe (CPTSSe) or CZTS (CZTSSe) was dissolved in 1 mL of isopropanol to form precursor inks, which were then sonicated to ensure effective dispersion of the nanoparticles and promote uniform film formation during deposition. After the substrates were prepared with a deposited and annealed CdS buffer layer, the kesterite absorber layer was deposited using a spin-coating technique. The sonicated kesterite nanoparticle inks were spin-coated onto the CdS buffer layer at 1500 rpm for 30 s, forming a thin film of the precursor on the substrate. The coated substrate underwent a thermal annealing process on a hot plate at 250 °C for 5 min in ambient air. To achieve the desired absorber thickness of approximately 800 nm, the spin-coating and heat treatment steps were repeated multiple times. No further heat treatment was applied after reaching the target thickness, as extensive characterization, such as X-ray diffraction (XRD), had already confirmed the successful formation of the kesterite phase in the nanomaterials. The kesterite absorber layer is vital for the performance of the PV device, as it absorbs incident sunlight, generates electron–hole pairs, facilitates charge-carrier transport to the electrodes, and determines the overall efficiency and performance of the device.

#### 2.2.4 Deposition of the Conductive Silver Paste Front Contact Layer

Conductive Ag paste, procured from Sigma-Aldrich, was used as the front contact. The paste was deposited onto the kesterite absorber layer to form the front contact electrode. The coated device was then placed on a hot plate preheated to 180 °C and subjected to a controlled thermal curing process for 5 min in ambient air to promote strong adhesion between the Ag paste and the underlying absorber layer, enhancing the charge-carrier collection efficiency. After curing, the device was allowed to cool to room temperature for thermal stabilization, ensuring consistent internal conditions during subsequent performance measurements. The Ag paste layer serves as the front contact, collecting photogenerated electrons and enabling current flow in the device.

#### 2.2.5 PV Efficiency (PCE) Measurements

After the thermal equilibrium was reached, the fabricated PV devices were ready for PV measurement to determine their device characteristics and PCE. The PCE is a key metric indicating the extent to which the fabricated devices can convert light energy into electrical energy. To obtain the device parameters for the prepared kesterite PV device, an Ossila source meter and accompanying software were used in conjunction with a Sciencetech SciSun solar simulator. The process was started by connecting the PV device to the Ossila source meter, ensuring that each of the 8 pixels of the PV device achieved proper contact for individual parameter measurements. The Sciencetech SciSun solar simulator was then calibrated to provide a consistent illumination intensity, typically set to AM1.5G standard conditions. The source meter, controlled by Ossila software, was programmed to perform current density–voltage (*J–V*) measurements across each pixel under illumination. These measurements were used to determine key device parameters, such as the short-circuit current density (*J*_sc_), open-circuit voltage (*V*_oc_), fill factor (FF), and PCE. The data collected by Ossila software provided detailed insights into the performance characteristics of the devices, allowing for the assessment of uniformity and identification of any performance-limiting factors within the devices.

### 2.3 Characterization

A collection of comprehensive characterization techniques was used to probe the physical, structural, and optoelectronic properties of the synthesized nanoparticles and fabricated kesterite devices. XRD analysis using a Malvern Panalytical Aeris diffractometer (Malvern Panalytical Ltd., Malvern WR14 1XZ United Kingdom) with Fe-filtered Co–Kα radiation (λ = 1.790300 Å) provided insights into crystal structure and phase formation. MATCH and DIAMOND software packages (Crystal Impact, Bonn, Germany) were used for further detailed crystallographic investigations and structural simulations. EdrawMax software was used to produce the graphical abstract (TOC). The surface morphology and elemental composition were investigated using a TESCAN MIRA’s third-generation scanning electron microscope (TESCAN ORSAY HOLDINGS, Brno-Kohoutovice, Czech Republic) equipped with energy-dispersive X-ray spectroscopy (EDS) capabilities. The low kilovolt (kV) imaging, low vacuum performance, and electron beam lithography features of this instrument enabled a thorough analysis of the surface textures and elemental distribution of the nanoparticles. Particle size and shape characterization was achieved through an Anton Paar small-angle X-ray scattering space (SAXSpace) instrument (Anton Paar GmbH, Graz, Austria). ATSAS software was used to evaluate the SAXS data and for Dummy atom reconstruction of the nanomaterials. 3D graphic representations were performed with UCSF ChimeraX (Resource for Biocomputing, Visualization, and Informatics at the University of California, San Francisco, United States of America). The optical properties, particularly the absorption characteristics within the 300–900 nm wavelength range at room temperature, were evaluated using a Varian Cary 300 ultraviolet–visible–near-infrared (UV–vis–NIR) spectrophotometer (Agilent, Santa Clara, CA, USA). Photoluminescence (PL) involving the excitation and emission of the nanomaterials was analyzed with a Horiba Nanolog Spectrofluorometer (Horiba Scientific, Minami-ku Kyoto, Japan). Electrochemical analyses, including differential pulse voltammetry (DPV) and electrochemical impedance spectroscopy (EIS), were performed using a CH Instrument electrochemical analyzer model CHI 760E (CH Instrument, Houston, Texas, United States of America) in a three-electrode configuration with a 0.071 cm^2^ glassy carbon disk working electrode, a platinum wire counter electrode, and a silver–silver chloride reference electrode. These measurements were conducted in 10 mL of 0.1 M tetrabutylammonium phosphate (TBAP)*−*acetonitrile electrolyte. Finally, the PV performance of the fabricated devices was assessed through *J–V* curve analysis using an Ossila Source Measure Unit with a Push-Fit Test Board (Ossila Ltd., Sheffield, United Kingdom) under AM1.5G illumination with 100 mW cm^–2^ irradiance provided by a SciSun (standard) low-cost solar simulator (Sciencetech Inc., London, Ontario, Canada). This comprehensive characterization strategy provided a critical understanding of the synthesized kesterite nanoparticles, paving the way for their optimization and integration into PV devices.

## 3 Results and Discussion

### 3.1 Morphological and Compositional Studies

**Figure 1:**
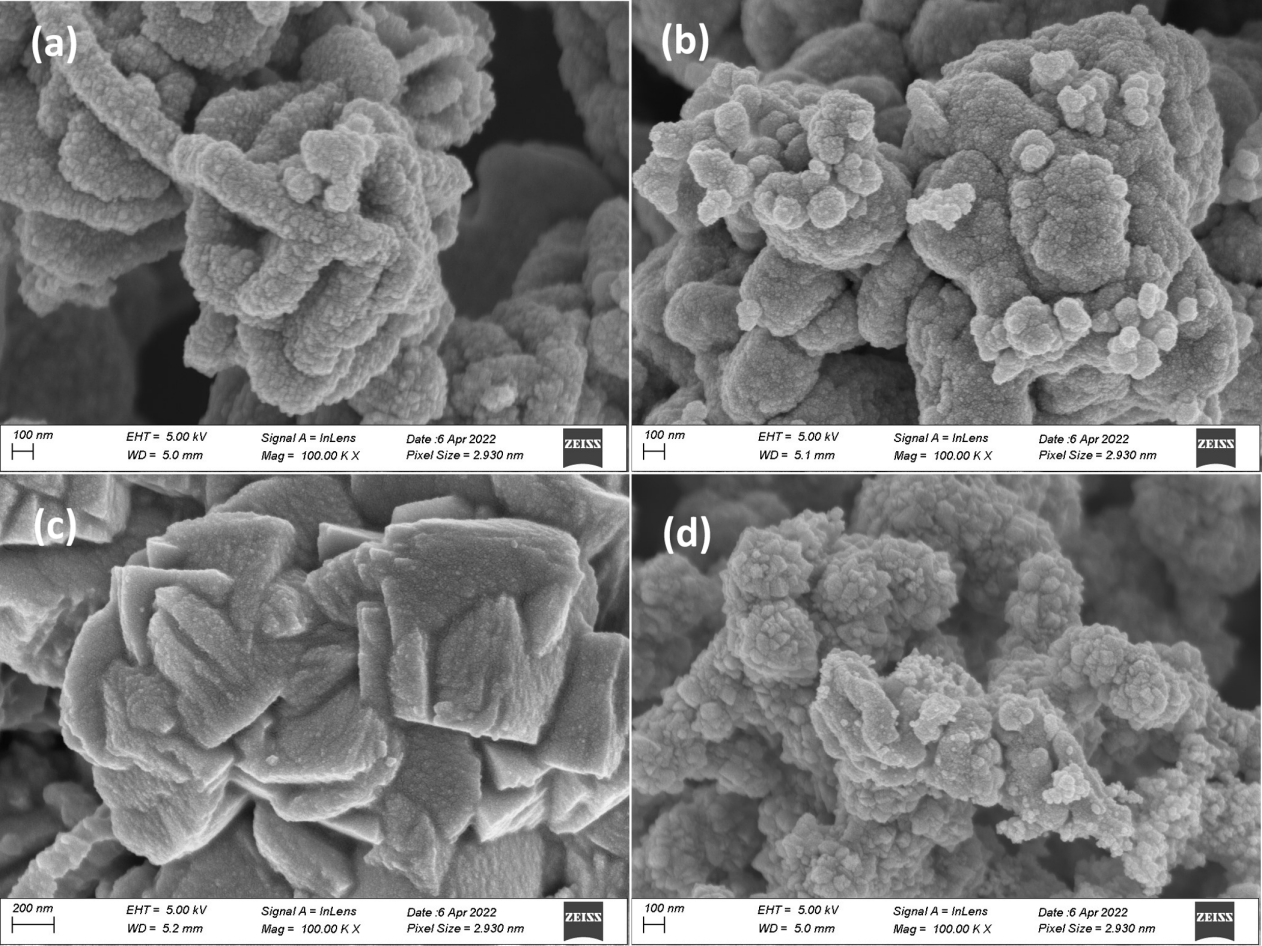
High-resolution scanning electron microscopy (HRSEM) images (with their scale) of (a) CZTSe, (b) CPTSe, (c) CZTSSe, and (d) CPTSSe nanoparticles.

The surface morphologies of the synthesized nanoparticles of CPTSe, CZTSe, CPTSSe, and CZTSSe were determined through HRSEM. Figure 1 displays the obtained HRSEM images of the nanomaterials. The CZTSe nanomaterial revealed a general flower-like morphology (Figure 1a). Polyshaped spherical particles can be seen from the micrographs obtained for the synthesized CZTSe material aligning in a cylindrical form. Figure 1c displays the morphology of CZTSSe, which also revealed a flower-like morphology with spherical-shaped particles aligning in a plate-like form. Figure 1b,d shows the morphologies of CPTSe and CPTSSe nanomaterials, respectively, which were nanospheres clustered to form a flower-like morphology. All of the synthesized nanomaterials clustered together forming larger-sized particles (agglomerated). Kesterite nanomaterials with a spherical shape and flower-like morphology have been reported to show good photocatalytic/photovoltaic properties.^23^ Generally, the flower-like morphology is attributed to the choice of the reaction solvent, where polyol solvents have been reported to yield nanomaterials with a spherical-shaped and flower-like morphology.^23^ The chemical composition of the nanomaterials was determined via EDS. Compositional deviations were noticed for CZTSe and CZTSSe nanomaterials, which particularly displayed low Zn and Sn contents as seen in the summarized atomic percent in Table 1. The loss of Zn can be attributed to the ease of formation of secondary phases such as ZnS as a result of the similarity of the atomic radii of Cu (145 pm) and Zn (142 pm) atoms.^13,24,25^ However, nanomaterials of CPTSe and CPTSSe conformed to the desired morphology, which can be attributed to the successful inhibition of secondary phase formation, which essentially is brought about by the larger difference in the atomic radii of Pd (169 pm) and Cu (145 pm) atoms by eliminating the formation of Cu_Zn_ antisites.^24,26^ Also, the formation of Cu_Pd_ antisite defects is inhibited as high energy is required for their formation^27^ due to the difference in the atomic radii of Cu and Pd atoms.^28^ The obtained chemical composition based on the atomic percent shows that the conditions of synthesis were favorable for the successful synthesis of Pd-substituted kesterite materials. This is made possible through the distinct chemical properties of Pd, which ensured the formation of kesterite compounds with the desired chemical composition, and by eliminating the possible formation of secondary phases as well as antisite defects.

**Table 1:** Elemental Compositions of CZTSe, CZTSSe, CPTSe, and CPTSSe Nanomaterials

sample	Cu (%)	Zn (%)	Pd (%)	Sn (%)	S (%)	Se (%)
CZTSe	21.46	0.13		1.38		77.03
CPTSe	23.43		16.95	13.87		45.75
CZTSSe	28.42	1.03		12.02	40.07	18.46
CPTSSe	27.60		14.74	11.79	34.06	11.81

### 3.2 Crystallographic and Structural Studies

**Figure 2:**
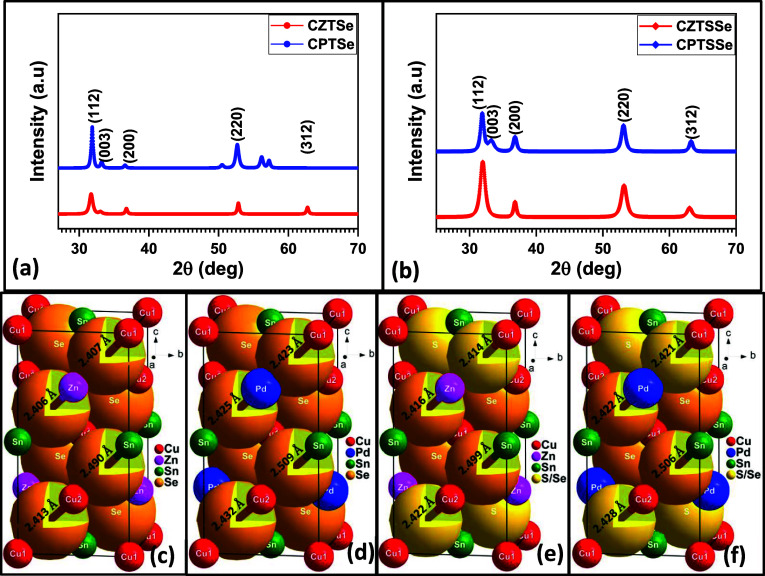
Powder XRD (PXRD) of (a) CZTSe, CPTSe and (b) CZTSSe, CPTSSe nanomaterials. Simulated crystal structures of (c) CZTSe, (d) CPTSe, (e) CZTSSe, and (f) CPTSSe nanomaterials.

The alloying and doping conditions affect the crystal structures of materials. The influence of elemental substitutions in synthesized materials can be evaluated through PXRD. The patterns of the synthesized nanomaterials are displayed in Figure 2. ICDD 26–0575 was used to index the observed peaks of the nanomaterials to the planes of the tetragonal kesterite phase. Figure 2a presents two PXRD patterns obtained from the analysis of CZTSe and CPTSe nanomaterials. CZTSe displayed peaks at 2θ values of 31.80°, 33.12°, 36.89°, 52.84°, and 62.76°, which were indexed to the planes of 112, 003, 200, 220, and 312, respectively. CPTSe patterns revealed peaks at 2θ values of 32.29°, 33.28°, 36.60°, and 52.63°, which were indexed to the planes of 112, 003, 200, and 220, respectively. Figure 2b presents the PXRD patterns of the CZTSSe and CPTSSe nanomaterials. CZTSSe displayed peaks at 2θ values of 31.98°, 36.82°, 53.17°, and 63.02°, which were indexed to the planes of 112, 200, 220, and 312, respectively. The CPTSSe nanomaterial displayed peaks at 2θ values of 31.89°, 33.28°, 36.82°, 53.10°, and 63.29°, which were indexed to the planes of 112, 003, 200, 220, and 312, respectively.^23,29−33^ All indexed peaks conform to the tetragonal kesterite phase configuration. The movement of the 2θ peaks to the left or right suggests lattice elongation or contraction, respectively, when materials are compared with each other.^34^ The patterns reveal a left shift of the peaks for CPTSe and CPTSSe nanomaterials at planes such as 112, 200, and 220, indicative of lattice elongation. This can be attributed to the expansion of the tetragonal lattice caused by the larger atomic radius of Pd. The left shift in the peaks of CPTSe and CPTSSe indicates successful incorporation of Pd atoms into the crystal lattice.^35^

The Debye–Scherrer equation is useful in determining the crystallite size distribution of the nanomaterials from the peaks obtained from their PXRD data, and it is expressed as follows
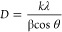
1where *D* is the crystallite size distribution, *k* is the shapeless factor with an approximate value of unity (0.9), θ is the peak positions in radians, λ is the wavelength of the source (1.7903 Å), and β_D_ is the full width at half-maximum (FWHM) broadening due to crystallite size (*D*), also in radians.

The *D* values of the nanomaterials are presented in Table 2 for CZTSe, CPTSe, CZTSSe, and CPTSSe nanomaterials, respectively. *D* distributions of 17, 22, 11, and 17 nm were obtained for CZTSe, CPTSe, CZTSSe, and CPTSSe nanomaterials, respectively. The use of Pd to replace Zn in the selenized kesterite materials resulted in an increased crystallite size. The larger-sized nanomaterials of CPTSe and CPTSSe can be attributed to the difference in the atomic radii of Zn and Pd, where Pd has a larger atomic size, thereby influencing the nucleation and growth rate of the nanomaterials.^36^

**Table 2:** Crystal Size Parameters for the Nanomaterials of CZTSe, CPTSe, CZTSSe, and CPTSSe

sample	2θ (deg)	*d*_(112)_ (Å)	FWHM (rad)	*D* (nm)
CZTSe	31.72	3.276	0.510	17
CPTSe	31.87	3.261	0.457	22
CZTSSe	31.97	3.248	0.776	11
CPTSSe	31.90	3.256	0.511	17

The indexing of the PXRD peaks to the kesterite phase indicates tetragonal crystal configuration of the nanomaterials.^2^ The crystal lattice parameters of the nanomaterials can be calculated by using the interplanar spacing (*d*_hkl_) formula for a tetragonal configuration as expressed in eq 2:
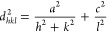
2where *h*, *k*, and *l* are the Miller indices, and *a*, *b* and *c* are the lattice constants. For a typical tetragonal crystal structure, the crystal lengths of the three edges of the unit cell are such that *a* = *b* ≠ *c*, and the angles in between the edges are such that *∝* = β = γ = 90°.^23^ The cell and crystal structural parameters are summarized in Table 3. From the lattice constant values obtained, the *a* value for the crystal lattice of the CPTSe nanomaterial was slightly higher than the *a* value of the CZTSe nanomaterial, while the *c* value was higher in CZTSe than in CPTSe. The higher *c* value obtained for the CZTSe nanomaterial resulted in its cell elongation compared to that of CPTSe, as seen from the volume of the unit cell (VUC) obtained for both nanomaterials. The VUC of a tetragonal crystal lattice can be calculated from the expression: VUC = *a*^2^*c*. The slight contraction in the crystal lattice of CPTSe shows that the incorporation of Pd did not drastically induce a left shift of all of the 2θ peaks. For the CPTSSe nanomaterial, the lattice constant values were higher when compared to the obtained values for the CZTSSe nanomaterial. The higher lattice constant values suggest the elongation of the crystal lattice^36^ of CPTSSe, which is confirmed by the VUC summarized in Table 2. Other notable crystal parameters are the atomic packing factor (APF) and the ratio of the lattice constant *c* to two times the lattice constant *a* (*c*/2*a*). The APF is a dimensionless entity that measures the volume fraction of the crystal lattice that is occupied by the constituent atoms, and its value is less than unity^37^ and can be calculated for tetragonal systems from the expression π*a*/3*c*. The *c*/2*a* ratio is a measure of the degree of tetragonal distortion that leads to the crystal field and nondegenerate valence band maximum.^38,39^ The *c*/2*a* value, which is less than 1, indicates that there is an increasing level of tetragonal distortion, which implies increased crystal field as well as a nondegenerate valence band maximum.^40^ As seen in Table 3, the APF values of CZTSe and CPTSe indicate that the constituent atoms of the CPTSe crystal lattice are slightly more tightly packed and occupy slightly more volume of their crystal lattice than the constituent atoms of CZTSe. The higher APF values indicate improved workability (ductility and malleability) of the CPTSe nanomaterial as its atoms are tightly packed, which leads to ease of atoms sliding over each other. On the other hand, the CZTSSe nanomaterial with a higher APF than the CPTSSe nanomaterial indicates improved workability for the nanomaterial. The *c*/2*a* obtained for the nanomaterials indicates that there is slight tetragonal distortion in the crystal lattice of the nanomaterials, with CPTSe showing the most distortion.^40^ The simulated crystal structures of the nanomaterials of CZTSe, CPTSe, CZTSSe, and CPTSSe are presented in Figure 2c–f, respectively. The bond distances between the metals and the chalcogens as seen from the simulated structures are slightly longer in the crystal lattices of CPTSe and CPTSSe. This bond-distance elongation can be attributed to the influence of Pd, which has an atomic/ionic radius larger than that of Zn. The larger atomic/ionic radius of Pd creates more distance in the crystal lattice, thereby resulting in the elongated bond distance. This is relatable to the crystallite size, as seen in Table 2.

**Table 3:** Crystal Structure Parameters for the Nanomaterials of CZTSe, CPTSe, CZTSSe, and CPTSSe

sample	*d*_(112)_ (Å)	*d*_(220)_ (Å)	*a* (Å)	*c* (Å)	*c*/2*a*	APF	VUC (Å^3^)
CZTSe	3.2760	2.0120	5.6908	11.2829	0.9913	0.5282	365
CPTSe	3.2606	2.0174	5.7061	11.0712	0.9701	0.5397	360
CZTSSe	3.2493	2.0003	5.6577	11.1397	0.9845	0.5319	357
CPTSSe	3.2580	2.0026	5.6642	11.2027	0.9889	0.5294	359

### 3.3 Statistical Studies of the Particle Shape and Size

**Figure 3:**
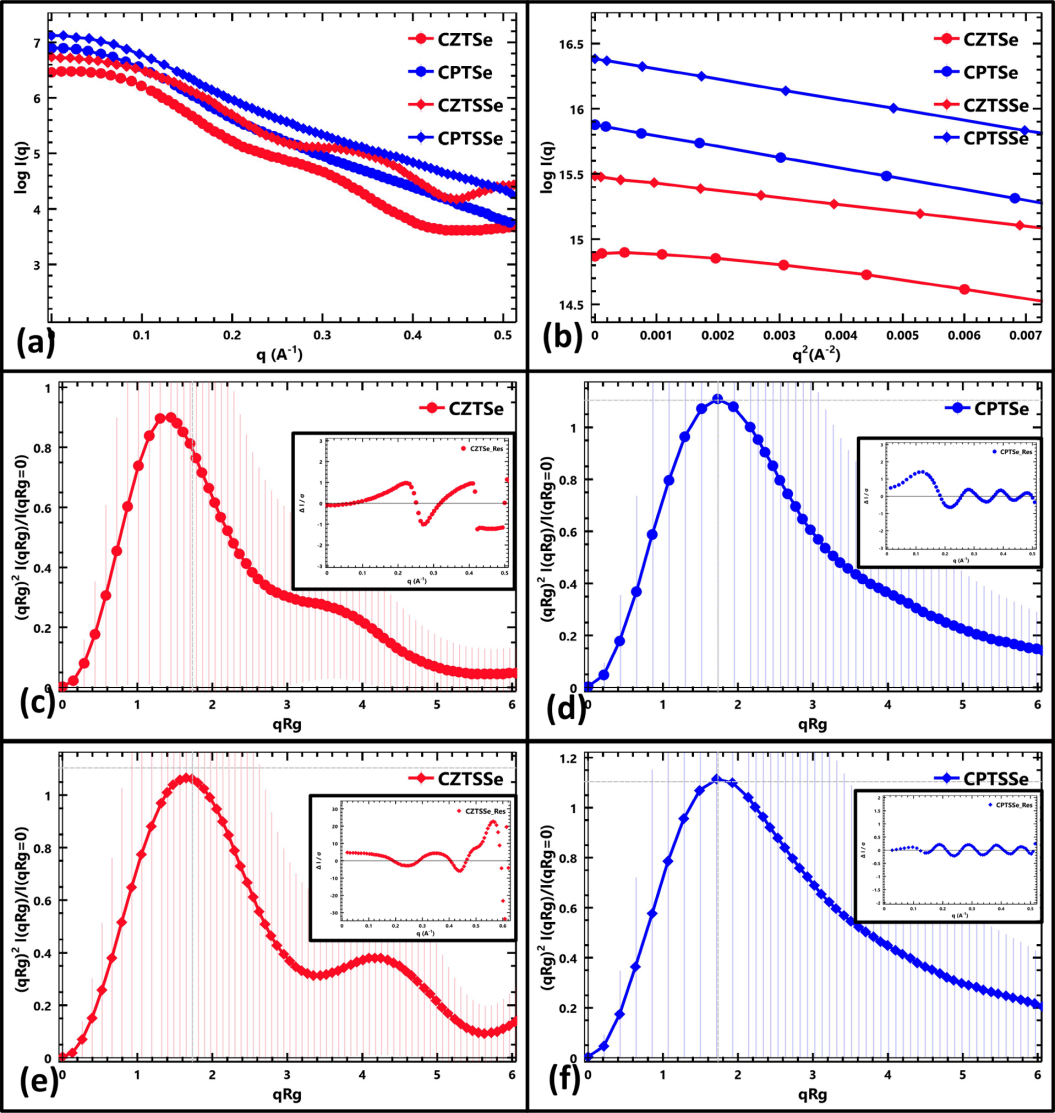
(a) Log–linear plots *I*(*q*) vs *q* of CZTSe, CPTSe, CZTSSe, and CPTSSe nanomaterials. (b) Guinier fits of CZTSe, CPTSe, CZTSSe, and CPTSSe nanomaterials. Dimensionless Kratky plots of (c) CZTSe, (d) CPTSe, (e) CZTSSe, and (f) CPTSSe nanomaterials. The insets display error-weighted residual plots of the nanomaterials.

SAXS is employed to characterize material structures on the nanometer to micrometer scale. X-ray irradiation of a sample generates a scattering pattern, with measurements focused on small angles (typically 0.1–10°). The resulting pattern reflects the size, shape, and internal arrangement of particles, pores, or other features within the material.^41^ Mathematical modeling and simulations are used to analyze the SAXS data, enabling the extraction of quantitative information regarding the nanostructure of the material.^42^ These analyses provide insights into material properties applicable in fields such as material science, biology, and nanotechnology. Data validation relies on various fundamental SAXS analyses, including log–linear/double-log plots (these visualize the raw scattering data, revealing features such as a high-*q* behavior or particle aggregation), Guinier plots (these plots provide information on the radius of gyration (*R*_g_), which provides information on the size and shape), *P*(*r*) plots (these plots depict the particle shape and size distribution), dimensionless Kratky plots (these plots offer insights into the overall particle structure), and Porod–Debye plots (these plots are used to estimate pore characteristics).^42^ The intensity of scattered X-rays reflects the atomic-level structure of the material. Figure 3a displays the log–linear plots of *I*(*q*) versus *q* for the investigated nanomaterials (CZTSe, CPTSe, CZTSSe, and CPTSSe). Notably, the plots for CPTSe and CPTSSe (Pd-based) exhibit a decay curve from the low-*q* to high-*q* regions. In contrast, the CZTSe and CZTSSe (Zn-based) plots also show a decay curve trend but with a distinct wave-like behavior in the high-*q* region (around 0.25 Å). This variation in the scattering patterns suggests that the Zn/Pd substitution influences the particle morphology of the nanomaterials. The intensity of the scattered X-rays is related to the electron density distribution within the material. A higher electron density translates into a higher scattered X-ray intensity in the low-*q* region. Figure 3a reveals that CPTSe and CPTSSe exhibit a stronger low-*q* intensity compared to their Zn counterparts. This observation suggests a higher electron density in the Pd-based materials, potentially due to differences in the atomic packing density. The low-*q* region intensity of scattered X-rays can also be indicative of the particle size. In Figure 3a, the higher intensity in the low-*q* region observed for CPTSe and CPTSSe suggests larger particle sizes compared to their Zn counterparts. Alternatively, the lower intensities observed for CZTSe and CZTSSe could be attributed to a higher concentration of vacancies or defects within their crystal structures. The presence of vacancies and defects disrupts the ordered atomic arrangement, leading to a lower scattering intensity. The intensity is also dependent on the atomic composition of the material. For instance, the higher scattering intensity of CPTSe compared to that of CPTSSe can be attributed to the higher atomic ratio of Se in CPTSe. Similarly, the higher intensity of CZTSe compared to CZTSSe in the low-*q* region can be explained by the differing Se content in these materials. As the atomic radius of Se is larger than that of S, nanomaterials with a higher Se content (CPTSe and CZTSe) will exhibit a higher low-*q* intensity, signifying larger average particle sizes compared to their double-chalcogenide counterparts. Based on the log–linear plots, CZTSe and CZTSSe are expected to have smaller average particle sizes compared to CPTSe and CPTSSe. Figure 3b presents the Guinier plots of the nanomaterials. A Guinier plot is a specific graph used to analyze the SAXS data. Information obtained from this plot is helpful in determining the size and shape of the nanomaterials.^42^ The plot is a logarithmic plot of the scattered intensity (ln(*I*) vs the square of the scattering vector (*q*^2^). A linear fit of the scattering data in the low-*q* region is used for the analysis. The slope of this linear region is related to the radius of gyration (*R*_g_) of the nanomaterials.^42,43^*R*_g_ is a measure of how far the mass of a particle is distributed from its center of mass. Larger *R*_g_ values indicate larger or more spread out particles.^42,43^ From Guinier plots, *I*(s = 0) can be estimated, which represents the intensity of the scattered radiation at zero scattering angle (s = 0). The value obtained for *I*(s = 0) can indicate the dense packing or concentration of the particles in the nanomaterials. Table 4 presents values obtained from the scattering data of the nanomaterials by using ATSAS primus software.

**Table 4:** Guinier and Pair Distance Distribution Function (*P*(*r*)) Analysis of the SAXS Data of the Nanomaterials of CZTSe, CPTSe, CZTSSe, and CPTSSe

sample	Guinier analysis		pair distance distribution function analysis				
	*R*_g_ (Å)	*I* (s = 0)	*R*_g_ (Å)	*I* (s = 0)	*D*_max_ (nm)	MW (Da)	*V*_p_ (Å^3^)
CZTSe	12.88	2,845,560	12.83	2,831,200	31	10,882	13,190
CPTSe	15.73	7,820,930	16.07	7,598,000	49	13,568	16445
CZTSSe	12.84	5,471,010	12.42	4,512,600	29	7936	9619
CPTSSe	15.40	13,025,300	15.45	12,783,000	47	10,975	13,306

**Figure 4:**
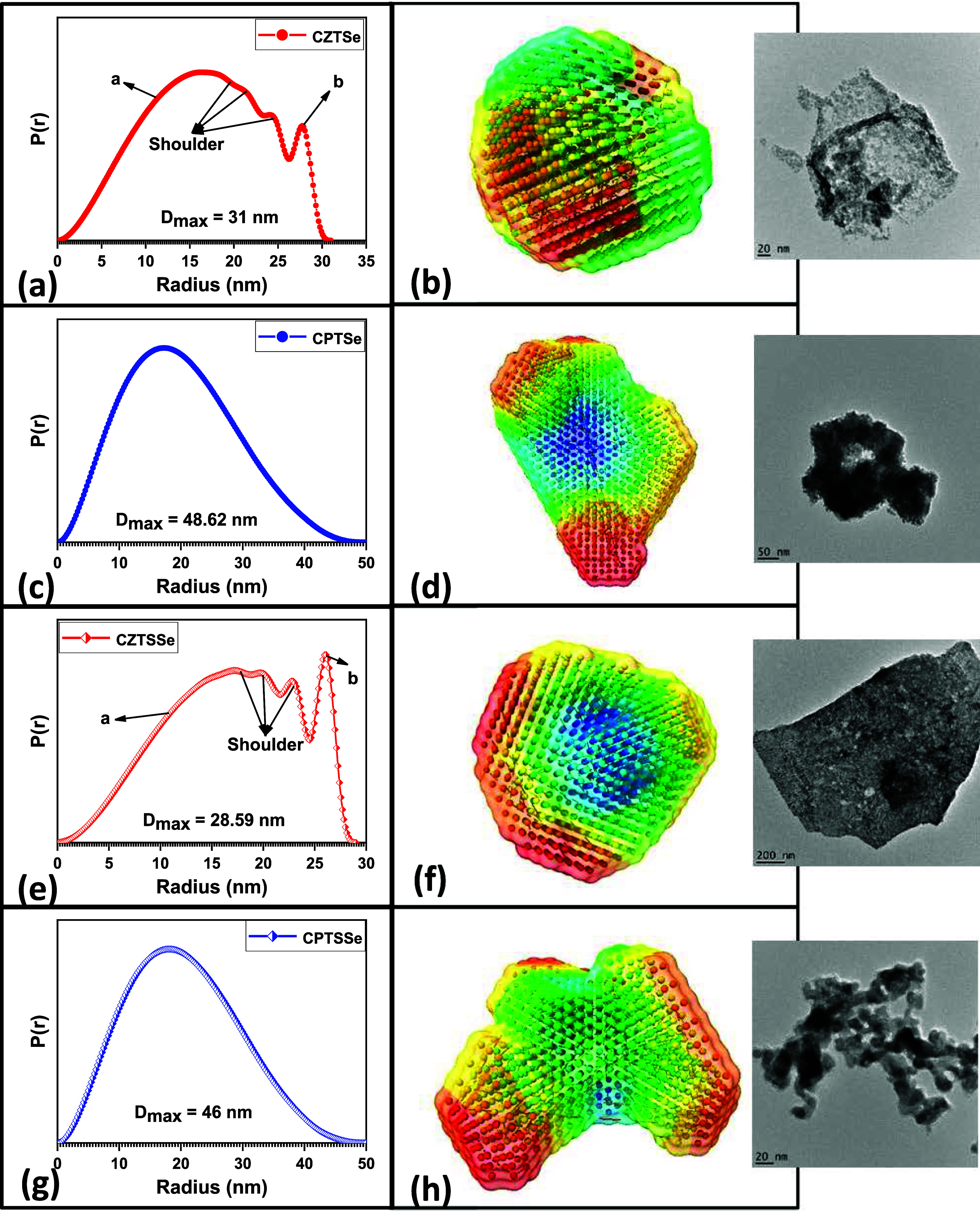
*P*(*r*) vs *r* profiles of (a) CZTSe, (c) CPTSe, (e) CZTSSe, and (f) CPTSSe nanomaterials. DAMMIF ab initio ball and stick shape simulations of (b) CZTSe, (d) CPTSe, (f) CZTSSe, and (h) CPTSSe nanomaterials, with insets showing their respective TEM images.

The *R*_g_ values obtained for the nanomaterials from the Guinier analyses are within the range of values expected from the analysis. *R*_g_ values of 12.88, 15.73, 12.84, and 15.40 Å were obtained for CZTSe, CPTSe, CZTSSe, and CPTSSe nanomaterials, respectively (see Table 4). It is observed that CZTSe and CZTSSe exhibit similar *R*_g_ values, which suggests that these nanomaterials have similar overall particle sizes or similar distributions of mass around their center of mass. On the other hand, there is a slight change in the *R*_g_ values of CPTSe and CPTSSe, indicating a slight difference in their particle size or the distribution of the mass around their center of mass. Generally, the higher *R*_g_ values of CPTSe and CPTSSe when compared to CZTSe and CZTSSe nanomaterials indicate that CPTSe and CPTSSe nanomaterials might have larger particles or may be more spread out in their mass distribution.^42^ The *I*(s = 0) values obtained for the nanomaterials (see Table 4) suggest that all nanomaterials contain a high concentration of scattering particles. The highest value obtained for CPTSSe suggests that it displays the highest concentration of scattering particles. It should be noted that there is no direct correlation between *R*_g_ and *I*(s = 0). The inset images in Figure 3c–f represent the error-weighted residual plots.^42,43^ For a good Guinier data analysis, the residuals should be randomly scattered around zero, especially in the low-*q* region. The plots for the nanomaterials show that their residuals were randomly scattered around zero, especially in the low-q region, suggesting that the Guinier plot provides a reasonable estimation. Some deviations are observed in CZTSe and CZTSSe in the higher *q* region, while CPTSe and CPTSSe maintained ordered random scattering around zero, which is good for the estimation of *R*_g_ during pair distance distribution function (*P*(*r*)) analysis, which requires the entire data range. The dimensionless Kratky plot is a SAXS analysis tool useful for understanding the overall shape and flexibility of particles. It offers a way to compare scattering profiles irrespective of the particle size or concentration. It reveals particle conformation in terms of a globular, folded, partly flexible, and unfolded appearance.^42−45^ In general, it offers information on the overall shape and flexibility of particles, revealing molecule folding and dynamics. At low *qR*_g_, the plot rises steeply, reaches a peak, and gradually decreases. Structures with folded domains show a bell-shaped curve with a maximum of 1.1 around a *qR*_g_ = 1.75. An increased elongation and degree of unfolding results in the maxima shifting to the upper right and an increase in the upward slope of the right side of the curve.^42^ The dimensionless Kratky plots of the nanomaterials are presented in Figure 3c–f for CZTSe, CPTSe, CZTSSe, and CPTSSe, respectively. CZTSe and CZTSSe nanomaterials display a more bell-shaped curve than CPTSe and CPTSSe nanomaterials. The maximum peak positions for the nanomaterials are 0.9 around a *qR*_g_ of 1.43, 1.07 around a *qR*_g_ of 1.64, 1.11 around a *qR*_g_ of 1.75, and 1.12 around a *qR*_g_ of 1.75 for CZTSe, CZTSSe, CPTSe, and CPTSSe, respectively. The information obtained shows that the nanomaterials conform to globularly shaped structures. The curve obtained for the nanomaterials shows that CZTSe and CZTSSe nanomaterials are folded particles, while CPTSe and CPTSSe are partially unfolded particles. It is also observed that the peak maxima for CZTSe and CPTSe were more compact than those for CZTSSe and CPTSSe nanomaterials.^42,43^ The curves for Pd-kesterites display more elongated particles than their Zn counterparts, as evidenced by the shifting of the peak maximum toward the right. The SAXS analytical technique can be used to determine the structure of nanomaterials in relation to their shapes or sizes.^46,47^ The *P*(*r*)) value of the SAXS data for the particle shape of materials should have a smooth concave approach to zero at *R* = 0. To obtain the *P*(*r*) profiles of the nanomaterials, the upper limit *D*_max_ was set at 35 nm for CZTSe and CZTSSe and 50 nm for CZTSSe and CPTSSe nanomaterials. At a *D*_max_ of 50 nm, the shape curve profiles of CZTSe and CZTSSe nanomaterials became distorted, suggesting that the limit is beyond the particle size range of the nanomaterials.^47^ At these upper limit *D*_max_ values, the *R*_g_ and *I*(s = 0) values obtained were relatable to the values obtained through the Guinier analysis, as reported in Table 4. The *D*_max_ (nm) values of the nanomaterials were 31, 48.62, 28.59, and 46 for CZTSe, CPTSe, CZTSSe, and CPTSSe, respectively. The *P*(*r*) profiles of the nanomaterials are presented in Figure 4. It is observable that the *P*(*r*) profiles of the Zn-based kesterite nanomaterials (Figure 4a,e) are similar. Also, the Pd-based kesterite nanomaterials (Figure 4c,g) exhibit similarity in their *P*(*r*) profiles. The inference here is that Zn and Pd have different effects on the shape of the kesterite materials. In the curve obtained for CZTSe and CZTSSe, two particle shapes are indicated and denoted as (a) and (b) in Figure 4a,e, respectively. The (a) part of these curves is associated with flat disk-shaped particles, while the (b) part of the curves is associated with long rod-shaped particles. This indicates that the particles associated with CZTSe and CZTSSe are polyshaped. Shoulders indicated in the curves of CZTSe and CZTSSe suggest particle aggregation or agglomeration.^22,47^ This explains the particle clusters observed in the HRSEM (Figure 1a,c) and HRTEM (inset of Figure 4b,f) images obtained for CZTSe and CZTSSe nanomaterials. The curves obtained for CPTSe and CPTSSe (Figure 4c,g) conform to solid spherical-shaped particles. The curves do not show any shoulders, indicative of lesser agglomeration. This is observed from the HRSEM (Figure 1b,d) and HRTEM (inset of Figure 4d,h) images. The shape plots from SAXS analysis decays to zero at the PDDF axis to a distance that represents the largest possible distance found inside the particles of the analyzed sample.^47^ These values indicate a slight particle size increase for CPTSe and CPTSSe nanomaterials, which is expected given the incorporation of the larger-sized atom of Pd into the kesterite structure.^36^ The influence of S and Se on the particle size of the nanomaterials can also be seen as CZTSe and CPTSe had larger *D*_max_ values than CZTSSe and CPTSSe, respectively, which can be attributed to the larger atomic radius of Se than that of S. Van der Waals forces often act on particles with very small particle sizes causing them to aggregate into larger particles.^48,49^ The aggregation seen by the shoulder in the shape plot of CZTSe and CZTSSe nanomaterials can be attributed to this phenomenon as the nanomaterial revealed a smaller particle size than CPTSe and CPTSe nanomaterials.

DAMMIF is a software program used for the ab initio (from first-principles) determination of the shape of a material from its SAXS data. Ab initio means measurement performed without any prior knowledge of the shape or atomic details of a material.^42,43^ DAMMIF uses mathematical models to generate shapes that would produce a scattering pattern similar to that of the experimental data. DAMMIF is a faster and more user-friendly version program called DAMMIN.^42^ The program works by taking the experimental SAXS data and generating a substantial number of random dummy atom models (usually represented as beads) within a defined search volume. For each model, the program calculates the theoretical scattering pattern that the model would produce. The program compares the theoretical scattering pattern from each model with the experimental data. The program then identifies the model that produces a theoretical scattering pattern that best fits the experimental data. These models can be considered as shapes of the molecules in the nanomaterials. DAMMIF software can be limited as the accuracy of the resulting models depends on the quality of the SAXS data.^42^ There are also situations where the program generates multiple models that fit the experimental data well. The ab initio models obtained for the experimental data of CZTSe, CPTSe, CZTSSe, and CPTSSe nanomaterials are presented in Figure 4b,d,f,h, respectively. The models obtained suggest that CZTSe and CZTSSe show globular cylindrical models, specifically hollow cylinders, while CPTSe and CPTSSe show globular ellipsoidal models, specifically an ellipsoidal model for CPTSe and a rotational ellipsoidal model for CPTSSe. The models generated were comparable to the *P*(*r*) profiles obtained for the materials, where CZTSe and CZTSSe nanomaterials exhibited polyshaped profiles of long rods and flat disks that are cylindrically shaped, and CPTSe and CPTSSe nanomaterials exhibited the profile of a solid sphere. Ellipsoidal shapes are spherical in shape. Comparing the models obtained from DAMMIF analysis with the images obtained from TEM analysis, we observed similarities between the appearance of the particles as seen in the TEM images and the fitted models from DAMMIF (see images in Figure 4b,d,f,h and their respective inset images).

Estimations of the molecular weight (MW) and porod volume (*V*_p_) of the nanomaterials were obtained from the experimental data. The MW of the nanomaterials are reported in Daltons (Da), and *V*_p_ are reported in cubic angstrom (Å^3^).^42,43,50,51^ The MW estimates and *V*_p_ were measured through *P*(*r*) analysis. The obtained values are reported in Table 4, where increasing MW and *V*_p_ values are observed in the sequence CZTSSe, CZTSe, CPTSSe, and CPTSe, respectively. This observed trend aligns well with the expected trend for the MW for the proposed chemical compositions of these nanomaterials, which was confirmed from EDS analysis.

**Figure 5:**
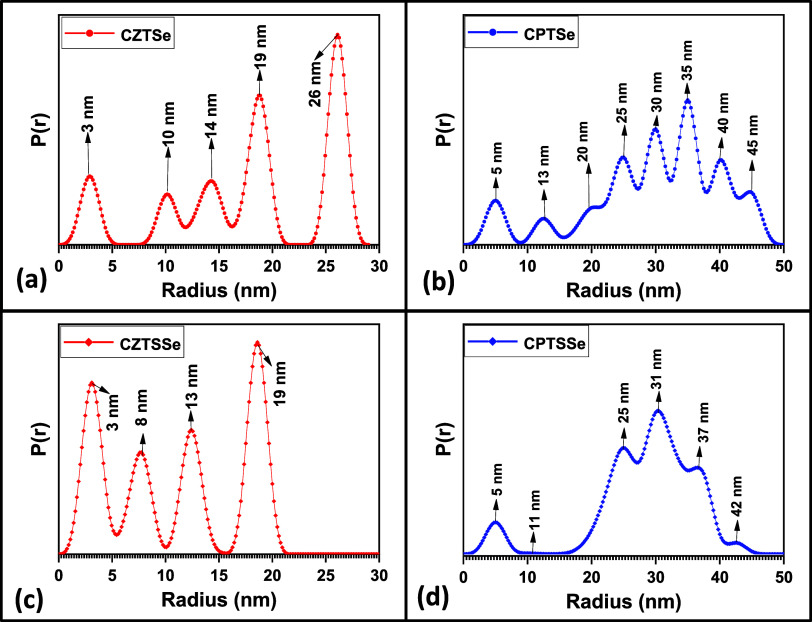
*P*(*r*) vs *r* profile of size plots of (a) CZTSe, (b) CPTSe, (c) CZTSSe, and (d) CPTSSe nanomaterials.

Figure 5 presents the *P*(*r*) vs *r* profile of the size distribution of the nanomaterials. It is observed that the particles of the nanomaterials are of varied sizes and hence polysized. Since CZTSe and CZTSSe are polysized and polyshaped, we can assume that their particles are polymorphous, while CPTSe and CPTSSe particles are polydispersed as they are only polysized. The particle size distribution obtained for CZTSe was 3–26 nm, CPTSe was 5–45 nm, CZTSSe was 3–19 nm, and CPTSSe was 5–42 nm. These values follow the same trend as the crystallite sizes obtained from XRD analysis, where crystallite sizes of 17, 22, 11, and 17 nm were obtained for CZTSe, CPTSe, CZTSSe, and CPTSSe nanomaterials, respectively. The particle size distribution obtained shows that the nanomaterials with Pd atoms had increased particle size in comparison to their Zn counterparts. This can be attributed to the atomic/ionic radius of Pd, which is larger than that of Zn, thereby affecting the nucleation and growth rates of the particles of CPTSe and CPTSSe.

### 3.4 Optical Studies

**Figure 6:**
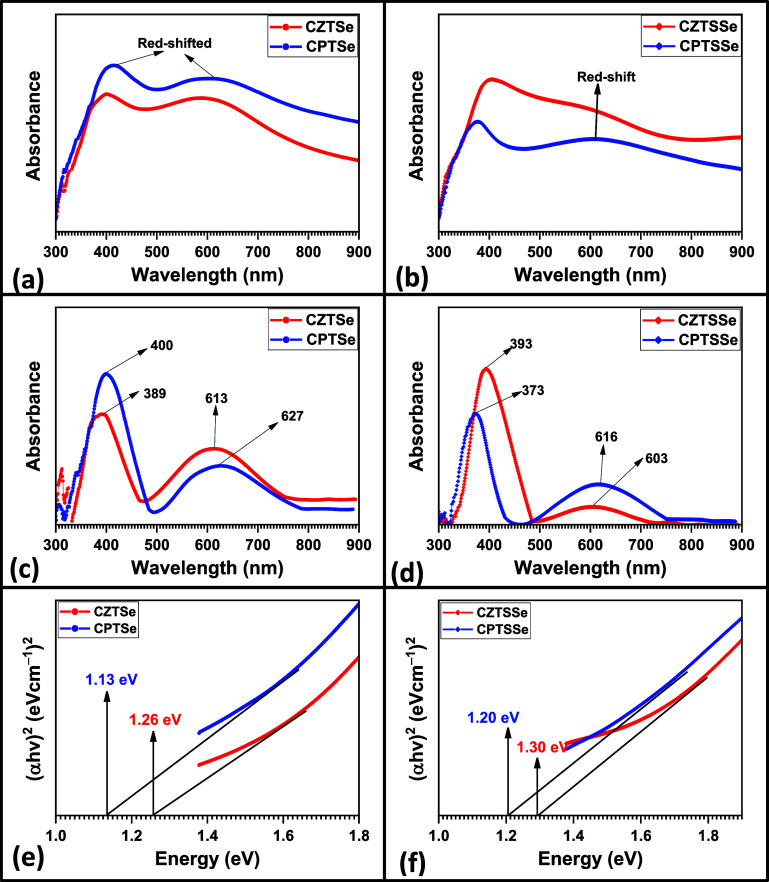
UV–vis spectra of (a) CZTSe, CPTSe and (b) CZTSSe, CPTSSe nanomaterials. Zero-baseline UV–vis spectra of (c) CZTSe, CPTSe and (d) CZTSSe, CPTSSe nanomaterials, revealing the wavelengths of their respective absorptions. Tauc plot of band gaps of (e) CZTSe, CPTSe and (f) CZTSSe, CPTSSe nanomaterials.

Optical properties of the synthesized nanomaterials were evaluated by UV–vis spectroscopy, and the obtained spectra are presented in Figure 6. The UV–vis spectra of the nanomaterials reveal optical properties influenced by their elemental composition. All four materials exhibit two distinct peaks: a sharp peak in the near-UV range around 380–400 nm and a broader peak in the visible range around 600–627 nm (see Figure 6c,d). These peaks can be attributed to the combined effect of band-to-band transitions and defect states within the materials.^52^ The near-UV peaks likely originate from intrinsic band-to-band transitions within the respective material's band gap, with slight variations in the peak position hinting at subtle differences in bandgap energies due to element substitutions (e.g., Cu, Zn vs Pd), indicating the potential for efficient light absorption in this region.^52−55^ The broader visible peaks suggest the presence of trap states or defect levels within the band gap, which can impact carrier transport and recombination processes.^52,53^ Subtle variations in peak positions and intensities are observed across the materials. CZTSe and CZTSSe, containing Zn, exhibit slightly blue-shifted UV peaks compared to CPTSe and CPTSSe, which might be due to differences in bandgap energies or defect states arising from the presence of Zn, and the slightly red-shifted peaks in CPTSe compared to CZTSe might be attributed to the presence of Pd, potentially affecting the band gap and light absorption. The broader visible peaks also show slight variations, indicating differences in the distribution or type of defect states within each material. The broader peak in CZTSSe and CPTSSe compared to the CZTSe and CPTSe could be due to the combined effect of sulfur and selenium, influencing the defect states and light-trapping capabilities. These observations suggest that tailoring the elemental composition of these nanomaterials could offer a route to manipulating their optical properties for optoelectronic applications.^46,56^ The bandgap values of the nanomaterials were obtained by converting the UV–vis spectra into a Tauc plot. The summary of the extrapolated bandgap values of the nanomaterials is presented in Table 5 and Figure 6c,d.

**Table 5:** Optical Properties of CZTSe, CPTSe, CZTSSe, and CPTSSe Nanomaterials

sample	*E*_g_ (eV)	*n*	ε_∞_
CZTSe	1.26	3.0427	9.2580
CPTSe	1.13	3.1267	9.7763
CZTSSe	1.30	3.0190	9.1144
CPTSSe	1.20	3.0801	9.4870

We can estimate the refractive index (*n*) property of optical materials from the Moss expression, *E*_g_*n*^4^ = *k*, where *E*_g_ is the extrapolated optical band gap, *n* is the refractive index, and *k* is a constant with a value of 108 eV. The *n* value reveals the speed of movement of light through an optical material. A high *n* indicates that an optical material can slow the speed of light that travels through it. This is especially important for PV materials for efficient photogeneration of carriers (electrons and holes).^57−60^ The *n* values of the synthesized nanomaterials are summarized in Table 5. The *n* values for CPTSe (3.1267) and CPTSSe (3.0801) when compared to CZTSe (3.0427) and CZTSSe (3.0190), respectively, show that the use of Pd atoms to supplant Zn atoms resulted in improved light retention properties of the kesterite materials, which resulted in continuous photogeneration.^57^ The dielectric properties of the kesterite materials were evaluated to determine their high-frequency dielectric properties.^61−63^ The dielectric properties refer to a ability of the material to impede electron flow, resulting in polarization when exposed to an external circuit. This means that they are able to store energy and produce electric fields with reduced energy loss.^51−53^ The high-frequency dielectric constant (ε_∞_) can be estimated from the expression ε_∞_ = *n*^2^*,* where *n* is the refractive index. The obtained dielectric constant values for the nanomaterials (see Table 5) reveal that the dielectric properties were improved in CPTSe and CPTSSe nanomaterials. This means that the energy storage of these nanomaterials was improved with the use of Pd to supplant Zn atoms. Pd-chalcogenide compounds have been reported to exhibit improved optical properties; this is the case for the novel nanomaterials of CPTSe and CPTSSe, showing improved optical properties compared to their Zn-kesterite counterparts. Also, the improved optical properties can be attributed to the difference in the atomic radii of Cu and Pd limiting the formation of secondary phases. The EDS spectra indicate that the use of Pd atoms to supplant Zn guaranteed a balanced compositional stoichiometry, while the use of Zn resulted in the loss of Zn atoms due to its instability during material synthesis conditions. The presence of defects and secondary phase formation are detrimental to the optical properties of kesterite materials as well as their overall light conversion performance.

**Figure 7:**
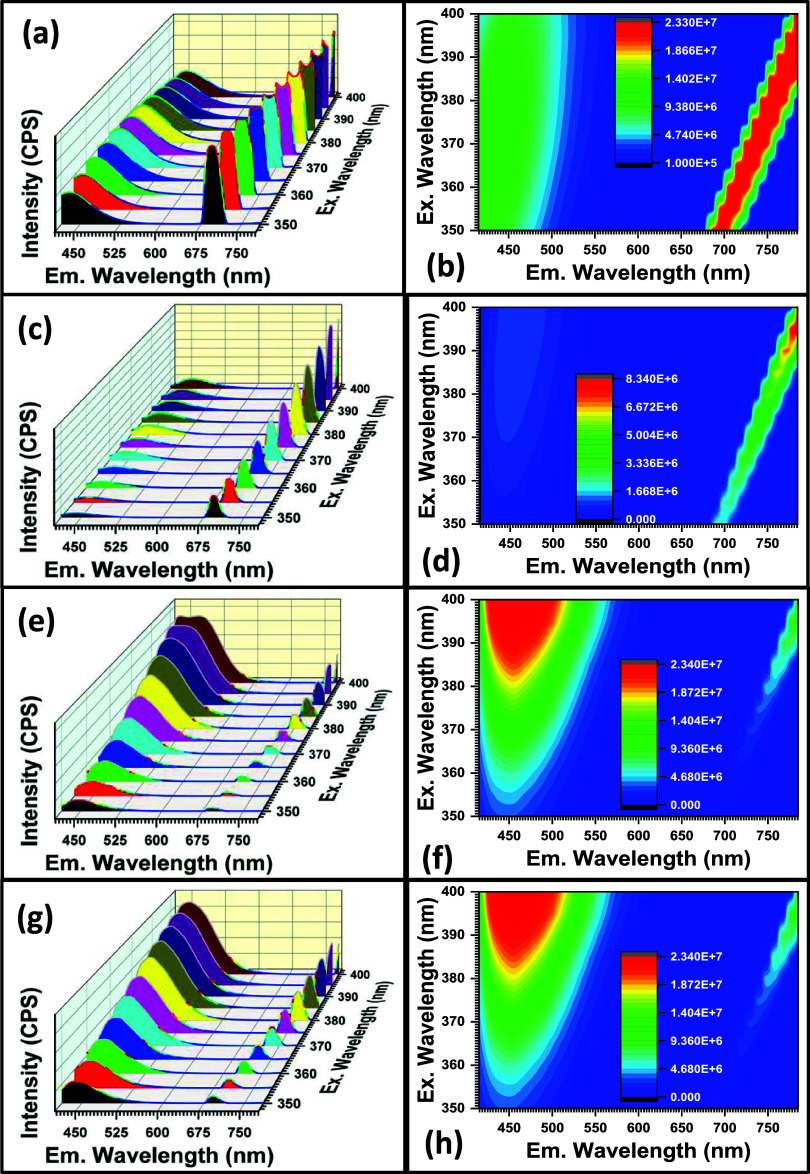
PL 3D spectra of (a) CZTSe, (c) CPTSe, (e) CZTSSe, and (g) CPTSSe nanomaterials. PL contour maps for (b) CZTSe, (d) CPTSe, (f) CZTSSe, and (h) CPTSSe nanomaterials (conditions: measured at excitation wavelengths of 350–400 nm).

PL spectroscopy is a spectroscopic technique that reveals the optical properties and crystalline defects of materials. PL in materials occurs due to the interaction of light with the electronic structure of the materials.^64^ The PL spectrum is a plot of the intensity of emitted light as a function of its wavelength. The specific characteristics of the PL spectrum, such as the peak position, intensity, and shape, depend on factors such as the composition, crystal structure, doping levels, defects, and environmental conditions (e.g., temperature) of the material.^65^ Analyzing the PL spectra of materials therefore provides insights into the properties and potential applications of the materials. The PL spectra of the nanomaterials were measured over several excitation wavelengths (from 350 to 400 nm with a 5 nm increase in between). The 3D PL spectra was used to present the emission behavior of the nanomaterials across the excitation wavelengths, as presented in Figure 7 alongside their PL contour maps. All four materials exhibit two main emission peaks around 450 and 680 nm. This suggests two dominant emission processes within the materials. The higher energy peak around 450 nm can be attributed to defect-related states, while the lower energy peak around 680 nm can be associated with the recombination of excitons at band edges.^52,53,65^ The peak at 680 nm was narrower and, in some cases, stronger than the peak at 450 nm. This indicates a higher probability of exciton recombination compared to emission from defect states in these materials under the given excitation conditions^52−54,66^ especially for CPTSe nanomaterials. The chemical composition of the nanomaterials influenced their emission. It is observed that the Zn counterparts exhibited emission peak splitting at certain excitation wavelengths, while the Pd counterparts did not show any peak splitting. Also, the use of S to replace Se in the nanomaterials enhanced the intensity of or favored one emission peak over the other, as seen in the spectra obtained for CZTSSe and CPTSSe. For the CZTSe nanomaterial (see Figure 7a,b), the behavior of the 450 nm peak suggests an interplay between defect states and excitonic recombination. The decrease in intensity at 380–400 nm excitation might be due to the saturation of defect states, while the overall increase with increasing excitation could be due to the high population of defect levels that contribute to the emission at 450 nm. The splitting of the 680 nm peak at higher excitation wavelengths could be due to the interaction of excitons with different phonon modes or vibrational states in the crystal lattice (in this case attributed to Zn). For the CPTSe nanomaterial (see Figure 7c,d), the weak and broad nature of the 450 nm peak suggests fewer defect states or weaker emission from them compared to CZTSe. This could be due to the presence of Pd instead of Zn, which influences the defect formation or recombination pathways. The strong and sharp 680 nm peak indicates efficient exciton recombination at the band edge of CPTSe. The strong and exponentially increasing intensity of the 450 nm peak suggests a high concentration of defect states, which contributes significantly to the PL in CZTSSe (see Figure 7e,f). The presence of S in the crystal lattice of CZTSSe creates more defect states or introduces new energy levels within the band gap, which favor emission at 450 nm. The splitting of the 450 nm peak at higher excitation wavelengths could be due to multiple defect states (linked to Zn) contributing to the emission. As in the case of CZTSSe, the strong 450 nm peak indicates a significant contribution from defect states in CPTSSe. The presence of S in this nanomaterial appears to have a more pronounced effect on defect-related emission. It can be inferred that in the case of CZTSSe and CPTSSe nanomaterials, the presence of S atoms favors emissions at 450 nm. This inference can be made due to the high intensity of the 450 nm peak, as observed from the obtained spectra. There was no peak splitting in the spectra of CPTSSe just like in CPTSe, suggesting some influence of Pd on the emission behavior of these nanomaterials. Overall deductions from the obtained spectra show that CZTSe has more defect states or defect configurations that favor emission at 450 nm compared to CPTSe. When comparing CZTSe and CZTSSe, the drastic increase in the intensity of the 450 nm peak with S incorporation underlines the sensitivity of defect-related emission to the material composition. Sulfur incorporation appears to create more favorable conditions for defect-related emission in CZTSSe.^22^ The same trend is observed for CPTSe and CPTSSe nanomaterials. The PL behavior of these materials is a fingerprint of their electronic structure and defect characteristics. The materials with efficient exciton recombination (680 nm peak) show suitability for solar cell applications.^22,28,67^ A PL color contour map is a visual representation of the intensity of light emitted from a material at different wavelengths, following excitation.^68^ It is a graphical tool to study the optical properties of the materials. In the map, the x and y axes typically represent different excitation or emission wavelengths, while the color intensity represents the relative intensity of the emitted light.^68^ Distinct colors correspond to different emission intensities. Hotter colors (typically red or yellow) represent regions of higher PL intensity, while cooler colors (typically blue or purple) represent lower intensity regions. The color contour maps obtained for the nanomaterials are presented in Figure 7b,d,f,h for CZTSe, CPTSe, CZTSSe, and CPTSSe, respectively. These color intensities support the trend reported for the PL properties of the nanomaterials.

### 3.5 Electrochemical Studies

**Figure 8:**
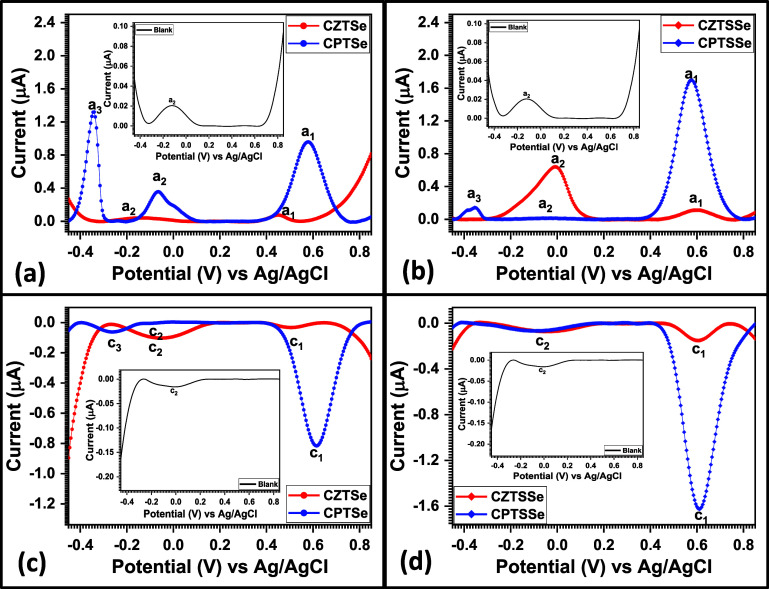
DPV overlays of bare GCE with (a) CZTSe, (b) CPTSe, (c) CZTSSe, and (d) CPTSSe nanomaterials (conditions: 0.1 M TBAP in acetonitrile at 50 mV s^–1^).

**Figure 9:**
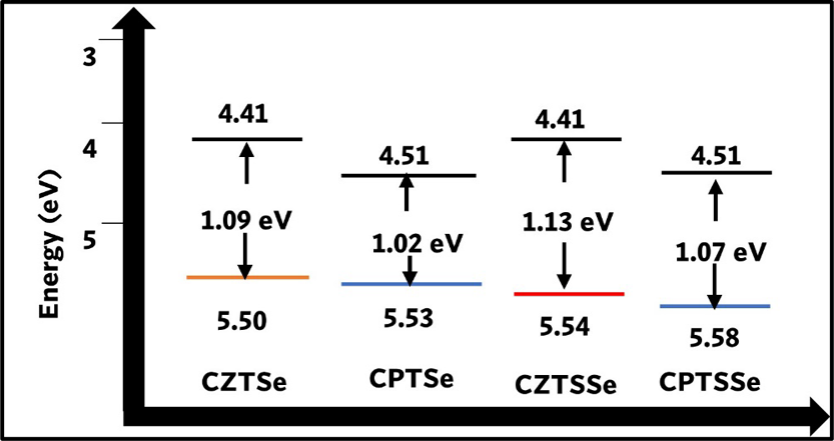
Energy band diagrams of (a) CZTSe, (b) CPTSe, (c) CZTSSe, and (d) CPTSSe nanomaterials obtained from DPV analyses.

Electrochemical properties of the nanomaterials were investigated via DPV due to the high sensitivity of the technique.^69^ The resultant voltammograms obtained for the nanomaterials are presented in Figure 8 at a scan rate of 50 mV s^–1^. 0.1 M TBAP in acetonitrile was used as an electrolyte under a three-electrode system consisting of a glassy carbon electrode as the working electrode, a platinum wire as the counter electrode, and a silver–silver chloride electrode as the reference electrode. The voltammogram obtained reveals oxidation and reduction peaks in the potential window used for the analysis (−0.45 to 0.85 V). On investigating the bare GCE electrode in TBAP, oxidation and reduction peaks were obtained and were marked as a_1_ and c_1_ and identified as the blank in all of the voltammograms plotted (see Figures 8a–9d). The anodic peak current (*i*_pa_) of the bare GCE (blank) in TBAP was 0.056 μA, with a corresponding anodic peak potential (*E*_pa_) of −0.145 V, and its cathodic peak current (*i*_pc_) was 0.02 μA, with a corresponding cathodic peak potential (*E*_pc_) of −0.0251 V. The ratio of *i*_pa_ to *i*_pc_ was approximately equal to 1, revealing an ideal reversible system.^70^ Its peak-to-peak separation (Δ*E*_p_) was −0.120 V with a formal potential (*E*°**′**) of −0.085 V.

The overall electrochemical properties of the nanomaterials via DPV are summarized in Table 6. The redox behavior of TBAP was observed in the DPV of all nanomaterials at the a_2_ and c_2_ peaks (see Figure 8). The redox behavior at a_1_ and c_1_ for all nanomaterials is due to the oxidation and reduction of copper.^71^ The oxidation peak at a_3_ observed for CPTSe and CPTSSe nanomaterials is attributed to the oxidation of palladium.^72^ The redox equations for a_1_ and c_1_ are given
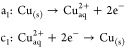
and the equation for a_3_ process is as follows
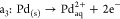


**Table 6:** Electrochemical Properties of CZTSe, CPTSe, CZTSSe, and CPTSSe Nanomaterials via DPV Analyses

sample	*i*_pa_ (μA)	*i*_pc_ (μA)	*E*_pa_ (V)	*E*_pc_ (V)	Δ*E*_p_ (V)	*E*°′ (V)
	a_1_, a_2_, a_3_	c_1_, c_2_,	a_1_, a_2_, a_3_	c_1_, c_2_	1, 2	1, 2
CZTSe	0.13, 0.12	0.20, 0.10	0.46, −0.14	0.50, −0.07	–0.04, −0.07	0.48, −0.11
CPTSe	0.94, 0.42, 1.30	0.82, 0.15	0.59, −0.07, −0.34	0.63, −0.08	–0.04, 0.01	0.61, −0.03
CZTSSe	0.21, 0.79	0.23, 0.11	0.63,–0.02	0.62, −0.01	0.01, −0.01	0.63, −0.02
CPTSSe	1.79, 0.06, 0.17	1.61, 0.11	0.58, −0.09, −0.36	0.62, −0.16	–0.04, 0.07	0.60, −0.13

The appearance of the peak at a_3_, which represents the oxidation of Pd, confirms the successful incorporation of Pd atoms in CPTSe and CPTSSe nanomaterials. The reduction in the peak current at a_3_ for the CPTSSe nanomaterial when compared to the same peak for CPTSe can be attributed to the atomic% of Pd atoms present in the material as revealed in the EDS analysis of the nanomaterials (see Table 1). The ratio of *i*_pa_ to *i*_pc_ for the redox system at a_1_ and c_1_ for the nanomaterial is approximately equal to 1, which reveals an ideal reversible system.^70^ The peak at a_3_ is an irreversible system (only seen in CPTSe and CPTSSe nanomaterials), and the redox systems at a_2_ and c_2_ reveal quasi-reversible systems as the ratio was more than 1. The reduction in Δ*E*_p_ particularly at a_2_ and c_2_ signifies the electrocatalytic properties of the nanomaterials as they were able to lower the energy barrier needed to oxidize and reduce TBAP ions.^73^ It was also observed that the peaks in this redox system (a_2_ and c_2_) were also enhanced, indicative of the electroconductivity of the nanomaterials. Overall, the highest *i*_pa_ values (μA) for the nanomaterials were 0.13, 1.30, 0.79, and 1.79 for CZTSe, CPTSe, CZTSSe, and CPTSSe, respectively. On the other hand, the highest *i*_pc_ values (μA) obtained for CZTSe, CPTSe, CZTSSe, and CPTSSe nanomaterials were 0.20, 0.82, 0.23, and 1.61, respectively. These peak current (*I*_p_) values show an improved electroconductivity in CPTSe and CPTSSe nanomaterials, with CPTSSe having the most improved electroconductivity. The band structure of nanomaterials can be evaluated via voltammetry techniques.^74^ Here, we obtained the electrochemical band gap *E*_g_ of the nanomaterials by taking the potential onset for the common oxidation peak a_2_ and reduction peak c_1_. The conduction band edge (LUMO) can be obtained from the reduction potential onset at c_1_, and the valence band edge (HOMO) can be obtained from the oxidation potential onset at a_2_. The electrochemical *E*_g_ can be evaluated from the expression *E*_g_ = *E*(LUMO)–*E*(HOMO), where *E*(LUMO) and *E*(HOMO) can be obtained from eqs 3 and 4, respectively.
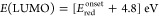
3
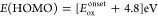
4

The energy band diagram of the nanomaterials is presented in Figure 9, and the summary of the parameters used for the estimation of the electrochemical band gap of the nanomaterials is presented in Table 7. The electrochemical *E*_g_ obtained followed the same trend as the optical *E*_g_, where CZTSSe > CPTSSe > CZTSe > CPTSe. The electrochemical *E*_g_ values (eV) of the nanomaterials were 1.09, 1.02, 1.13, and 1.07 for CZTSe, CPTSe, CZTSSe, and CPTSSe nanomaterials, respectively. The reduced *E*_g_ values for CPTSe and CPTSSe nanomaterials indicate that they will exhibit improved light absorption and photogeneration of carriers, which will lead to enhanced PV performance.

**Table 7:** Electrochemical Bandgap Parameters of CZTSe, CPTSe, CZTSSe, and CPTSSe Nanomaterials from DPV Analyses

sample	*E*_ox. onset_ (V)	*E*_red. onset_ (V)	HOMO (eV)	LUMO (eV)	*E*_g_ (eV)
CZTSe	–0.39	0.70	4.41	5.50	1.09
CPTSe	–0.29	0.73	4.51	5.53	1.02
CZTSSe	–0.39	0.74	4.41	5.54	1.13
CPTSSe	–0.29	0.78	4.51	5.58	1.07

**Figure 10:**
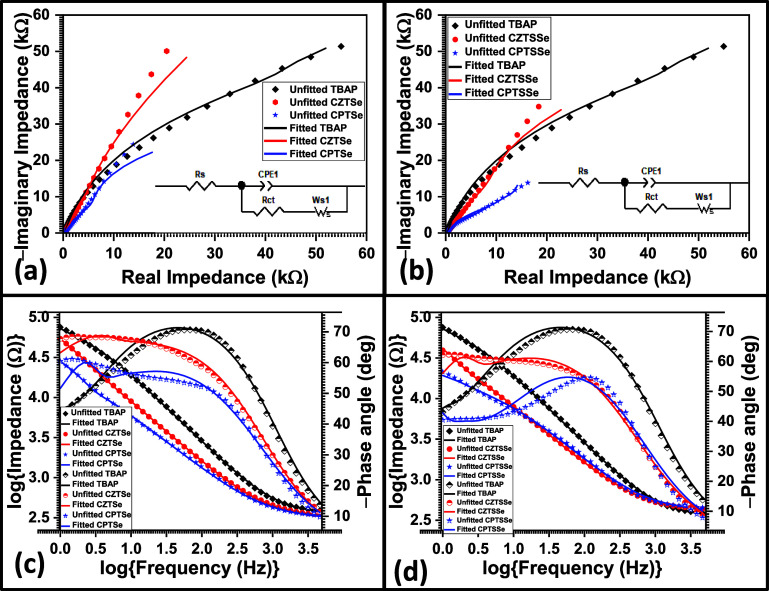
Nyquist plot overlay of bare GCE with (a) CZTSe and CPTSe and (b) CZTSSe and CPTSSe nanomaterials. Bode plot overlay of bare GCE with (c) CZTSe, CPTSe and (d) CZTSSe and CPTSSe nanomaterials (conditions: 0.1 M TBAP in acetonitrile).

**Figure 11:**
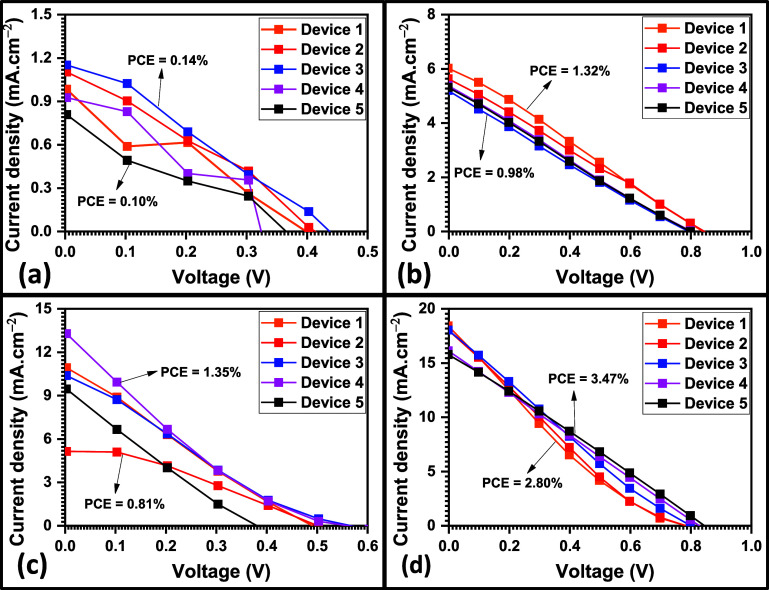
*JV* curves of (a) CZTSe, (b) CPTSe, (c) CZTSSe, and (d) CPTSSe PV devices (five devices based on each nanomaterial were fabricated and evaluated).

Electrokinetics of the nanomaterials were evaluated via EIS. This experiment was conducted under the same conditions as those used for DPV analyses. EIS analyses involve two distinctive plots (the Nyquist plot and the Bode plot), which relay the same electrokinetic properties of the material under study. The Nyquist plot involves the relation of imaginary impedance to real impedance, and extrapolation of the semicircle to the *x*-axis gives the charge-transfer resistance (*R*_ct_) of the material being studied in the high-frequency region.^75^ The Bode plot relays the same information by comparing the logarithm of the magnitude of impedance and the negative phase angle to the logarithm of frequency. The magnitude of impedance is evaluated at the lower region of the Bode plot.^22^ The data obtained from the analysis was fitted with ZView to produce the equivalent circuit of the electrochemical systems of the nanomaterials. All nanomaterials fitted the same equivalent circuit, which is shown as an inset in the Nyquist plots presented in Figures 10a and 11b. The obtained electrokinetic properties of the nanomaterials are summarized in Table 8.

**Table 8:** EIS Parameters of CZTSe, CPTSe, CZTSSe, and CPTSSe Nanomaterials

sample	*R*_s_ (kΩ)	*R*_ct_ (kΩ)	–ϕ_peak_ (deg)	*v*_peak_ (Hz)	*Z*_mag_ (kΩ)
GCE	347	82	71	68	73
CZTSe	314	60	66	18	54
CPTSe	295	28	57	22	28
CZTSSe	395	56	61	20	39
CPTSSe	405	14	55	56	21

For comparison, the spectra obtained for the nanomaterials and the bare GCE in TBAP were overlaid and are presented in Figure 10. The Randles–Sevcik equivalent circuit (inset in Figures 10a and 11b) of the nanomaterials consisted of the ohmic resistance (*R*_s_), which emanates from resistance due to the electrolyte, *R*_ct_, which is the resistance attributed to the interfacial interaction of the electrolyte and the electrode surface modified with the nanomaterials, the constant phase element (CPE), which is due to the inhomogeneity of the electrode surface, and a short Warburg (*W*), which is due to ionic diffusion of the modified electrodes.^75^ In Table 8, the bare GCE in TBAP had an *R*_ct_ of 82 kΩ, and on modification of the surface with the nanomaterials, the *R*_ct_ was drastically reduced. *R*_ct_ values of 28, 14, 60, and 56 kΩ were obtained for CPTSe, CPTSSe, CZTSe, and CZTSSe nanomaterials, respectively. This outcome correlates with the current peaks obtained for the same materials as seen from DPV analyses. The reduced *R*_ct_ obtained when the GCE was modified with the nanomaterials implies that the interfacial energy barrier between the electrode and the electrolyte were reduced, resulting in enhanced mobility and diffusion of electrons in the electrochemical systems.^22^ The replacement of Zn atoms with Pd atoms improved the electrochemical kinetics of the kesterite species, as evidenced by their reduced *R*_ct_ values. The reduced *R*_ct_ of CPTSe and CPTSSe nanomaterials implies enhancement of the surface area of the parent kesterites, resulting in improved electrocatalysis and electroactivity. The *R*_ct_ value was the lowest for the CPTSSe-modified electrode, which correlates with the highest peak current value obtained for the same material as seen from the DPV analysis.

The active surface sites (θ) of the modified electrodes can be estimated from *R*_ct_ from the expression
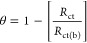
5where *R*_ct(b)_ is the charge-transfer resistance due to the bare electrode, and *R*_ct_ is the charge-transfer resistance due to the modified electrode. With a maximum value of 1, θ indicates the extent of surface area activation by electroactive materials. The θ values obtained were 0.27, 0.66, 0.32, and 0.83 for CZTSe-, CPTSe-, CZTSSe-, and CPTSSe-modified electrodes, respectively. This implies that 27% more active sites were activated by using CZTSe nanomaterial to modify the electrode surface of bare GCE. The same follows that 66% new active sites were formed for bare GCE modification with CPTSe, as well as 32% new active sites were induced using CZTSSe, and finally 83% more active sites emanated from the electrode surface modification with CPTSSe. The use of Pd to replace Zn therefore improved its electrochemical properties and induced an improved surface conductivity of the material. This correlates with the analyses from the zeta potential discussed earlier. Although Zn is more electrically conductive than Pd,^76^ yet the ease of loss of the atom during synthesis results in poor electrochemical properties while the use of Pd to replace Zn atoms ensures the formation of the compound in the correct stoichiometry, and few or no atoms of Pd are lost during synthesis, which therefore ensures the stability of the material and enhances their electrochemical properties as Pd is also an element with good electrical conductivity.

The apparent (*k*_app_) and real (*k*_0_) rate constants of the electrochemical systems^77^ were evaluated from the expressions in eqs 6 and 7, respectively.
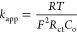
6
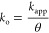
7where *R* is the gas constant, *T* is the temperature, *F* is the Faraday constant, *R*_ct_ is the charge-transfer resistance of the electrode system, and *C*_0_ is the concentration. The *k*_app_ values obtained were 4.43 × 10^–8^, 9.50 × 10^–8^, 4.75 × 10^–8^, and 19.0 × 10^–8^ cm s^–1^ for CZTSe, CPTSe, CZTSSe, and CPTSSe nanomaterial-modified electrode systems, respectively, with their corresponding *k*_0_ values of 16.4 × 10^–8^, 14.4 × 10^–8^, 14.8 × 10^–8^, and 22.9 × 10^–8^ cm s^–1^, respectively. CPTSSe with the highest *k*_app_ and *k*_0_ indicates faster electrokinetics, which correlates with the obtained high current peaks from DPV analyses.

Bode plots presented in Figure 11c,d were used to analyze the electrochemical behavior of the GCE electrode modified with the synthesized nanomaterials in relation to the magnitude of impedance (*Z*_mag_) in the low-frequency (*v*) region and the peak frequency (*v*_peak_) due to the peak phase angle (−ϕ_peak_). The obtained values are summarized in Table 8 and indicate improved electrochemical kinetics with nanomaterial modification of the GCE. The electrocatalytic properties of the material can be seen in the lowering of the ϕ of bare GCE (−71°), with GCE modified with CPTSSe showing the most reduced ϕ value of −55°. In comparison to CZTSe (−66°), the CPTSe-modified GCE also revealed a lower ϕ value of −57°. The lowered ϕ values for CPTSe- and CPTSSe-modified GCEs indicate that the use of Pd atoms to supplant Zn atoms improved the kesterite electrocatalytic properties as the lowered ϕ value indicates that the activation energy barrier of the electrode system is lowered to readily speed up the electrochemical reaction, enhancing the electron mobility of the system.^78,79^ It was observed that the *v*_peak_ of the bare GCE was obtained in the higher *v* region (68 Hz), suggesting a tendency for faster charge-transfer reactions.^80−85^ CPTSe and CPTSSe, which had *v*_peak_ values of 22 and 56 Hz, respectively, displayed faster charge-transfer reactions than their counterparts CZTSe and CZTSSe nanomaterials (with *v*_peak_ values of 18 and 20 Hz, respectively). In relation to the magnitude of impedance in the electrode/electrolyte system, the modified electrodes displayed reduced *Z*_mag_ values of 54, 28, 39, and 21 kΩ for CZTSe, CPTSe, CZTSSe, and CPTSSe nanomaterials, respectively, when compared to a *Z*_mag_ value of 73 kΩ obtained for bare GCE in the lower *v* region. The *Z*_mag_ observed in CPTSe and CPTSSe electrode/electrolyte interfacial interactions implies that the nanomaterials reduced the total impedance to current flow in the electrochemical systems. This observation correlates with the reduced *R*_ct_ value obtained from the Nyquist plot and supports the improved current peak values observed from DPV analyses of the same electrode systems, especially for the CPTSSe-modified electrode.

### 3.6 PV Studies

The CZTSe, CPTSe, CZTSSe, and CPTSSe nanomaterials were converted into nanoinks and applied as absorber layers to construct their PV devices. The superstrate PV architecture of the devices was ITO-coated glass/Al:ZnO/*i*-ZnO/CdS/kesterite layer/Ag-paste. Dissolved inks of each layer were coated using a spin-coating technique. Following their fabrication, the devices were evaluated to determine their PCE. To determine the PCE from light energy to electrical energy, the short-circuit current density (*J*_sc_) generated in the device and their open-circuit voltage (*V*_oc_) values were measured. The current density–voltage (*JV*) curve plot provides details about the PV characteristics of the devices, which were used to estimate their PCEs.

Figure 11 presents the *JV* curves for the fabricated kesterite PV devices. Table 9 summarizes the PV parameters obtained for the highest-performing devices and compares them to other kesterite devices reported in the literature, fabricated by using a superstrate architecture.^82−85^ The device measurements were conducted with a solar simulator under 100 mW cm^–2^ AM1.5G illumination. Figure 11a depicts the *JV* curves for CZTSe-based devices under illumination. Five devices were fabricated for each kesterite PV type. The CZTSe devices exhibited low cell parameter values, with PCEs ranging from 0.10 to 0.14%. The *J*_sc_ and *V*_oc_ values ranged from 0.8 to 1.11 mA cm^–2^ and 0.32 to 0.43 V, respectively. The fill factor (FF*)* values for the CZTSe devices varied from 24.75 to 40.81%. Figure 11b shows the *JV* curve of CPTSe-based devices, which outperformed the CZTSe devices, with PCEs ranging from 0.98 to 1.32%, approximately ten times higher than those of the CZTSe-based devices. The *J*_sc_ values for CPTSe-based devices ranged from 5.2 to 6.0 mA cm^–2^, while the V_oc_ values ranged from 0.78 to 0.84 V. FF values for CPTSe-based devices were between 24.92 and 27.79%. The *J*_sc_ and *V*_oc_ values were double those of the CZTSe-based devices. Figure 11c displays the performance of the CZTSSe-based devices. The PCEs ranged from 0.81 to 1.35%, with improved *J*_sc_ and *V*_oc_ values ranging from 5.5 to 14.2 mA cm^–2^ and 0.36 to 0.59 V, respectively. FF values for CZTSSe-based devices ranged from 20.14 to 31.71%. Figure 11d illustrates the performance of CPTSSe-based devices. The PCEs ranged from 2.80 to 3.47%, with enhanced *J*_sc_ and *V*_oc_ values from 15.40 to 18.03 mA cm^–2^ and 0.79 to 0.85 V, respectively. Although CZTSSe showed improved *J*_sc_ values, CPTSSe-based devices performed better overall, particularly with *V*_oc_ and PCE values double those of CZTSSe devices. *FF* values for CPTSSe-based devices ranged from 20.07 to 26.72%. These results indicate that Pd-based devices outperform their Zn-based counterparts. This can be attributed to reduced crystal defects, optimal chemical composition, and improved optical properties. Notably, *V*_oc_ improvements were observed in CPTSe and CPTSSe devices, which can be attributed to the reduction of the detrimental Cu_Zn_ antisite defect as a consequence of the substitution of Zn atoms with Pd atoms. The deficient performance of CZTSe-based devices is likely due to high crystal defect levels observed in PL analyses and their suboptimal chemical composition, evidenced by the significant loss of Zn and Sn atoms. Additional factors may include thin-film inhomogeneity during device fabrication.

**Table 9:** Comparison of the Fabricated PV Devices with Other Reported Kesterite Devices Fabricated via Superstrate Architecture

device architecture	*V*_oc_ (mV)	*J*_sc_ (mA cm^–2^)	FF (%)	PCE (%)	ref
FTO/TiO_2_/CZTSe_3.2_S_0.8_/Spiro/Ag	290	36.5	38.00	3.10	(82)
ITO/CdS/CZTSSe/Au	77	2.45	25.00	0.10	(83)
ITO/TiO2/CdS/CZTSSe/Au	363	6.83	44.70	1.10	(84)
ITO/CdS/CZTSe/Carbon	433	13.72	41.10	2.44	(85)
ITO/Al:ZnO/*i*-ZnO/CdS/CZTSe/Ag	433	1.11	28.87	0.14	this work
ITO/Al:ZnO/*i*-ZnO/CdS/CPTSe/Ag	841	6.00	27.79	1.33	this work
ITO/Al:ZnO/*i*-ZnO/CdS/CZTSSe/Ag	548	11.12	22.99	1.40	this work
ITO/Al:ZnO/*i*-ZnO/CdS/CPTSSe/Ag	845	15.40	26.73	3.47	this work

**Figure 12:**
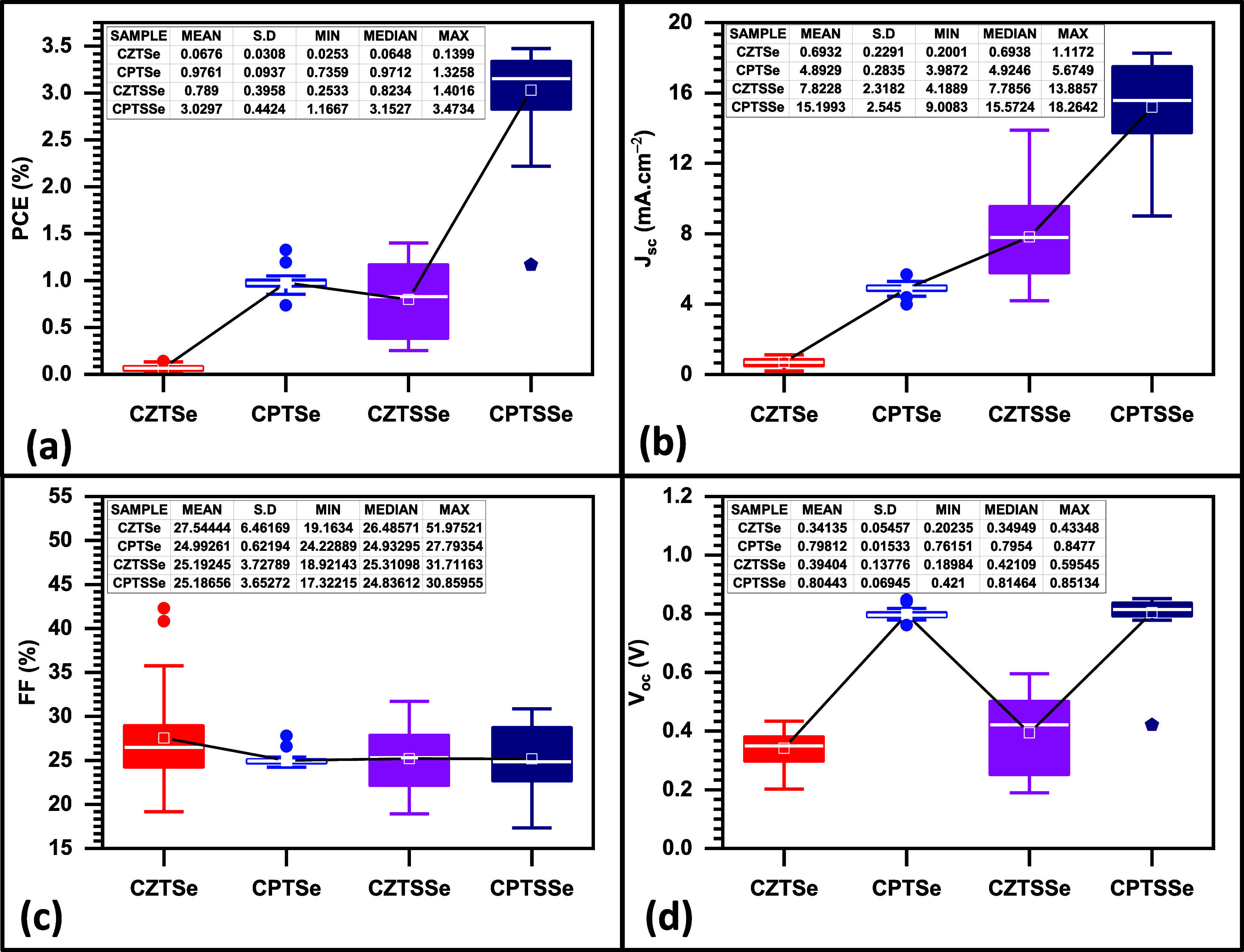
Statistical PV performance of the best CZTSe-, CPTSe-, CZTSSe-, and CPTSSe-based devices by (a) PCE, (b) *J*_sc_, (c) FF, and (d) *V*_oc_ (evaluation performed for 24 days; the inset table shows the mean, standard deviation (SD), minimum, median, and maximum values of the parameter obtained for each device based on the nanomaterials).

Figure 12 illustrates the statistical performance parameters of the highest efficiency devices based on CZTSe, CPTSe, CZTSSe, and CPTSSe nanomaterials, evaluated over a 24 day period. The average PCEs were found to be 0.07, 0.98, 0.79, and 3.03% for devices based on CZTSe, CPTSe, CZTSSe, and CPTSSe, respectively (see Figure 12a and the inset tables). Correspondingly, the mean *J*_sc_ values were 0.69, 4.89, 7.82, and 15.20 mA cm^–2^ for CZTSe, CPTSe, CZTSSe, and CPTSSe devices, respectively (see Figure 12b). The average FF values were recorded as 27.54, 24.99, 25.19, and 25.19% for CZTSe, CPTSe, CZTSSe, and CPTSSe-based devices, respectively (see Figure 12c). The mean *V*_oc_ values were measured to be 0.34, 0.80, 0.39, and 0.80 V for CZTSe, CPTSe, CZTSSe, and CPTSSe, respectively (see Figure 12d). Over the 24 day monitoring period, the CZTSe and CZTSSe devices deteriorated by ∼80% of their initial performance values, whereas the CPTSe and CPTSSe devices deteriorated by ∼30%. This indicates a substantial enhancement in carrier lifetime and efficiency when Pd atoms replace Zn atoms in kesterite materials and devices.

The CPTSe- and CPTSSe-based devices displayed the best device properties, with the CPTSSe-based device performing the best with a PCE of 3.47% and the CPTSe device displaying a PCE of 1.33%. Also, device parameters such as *J*_sc_ and *V*_oc_ were improved in these materials when compared to the Zn-based kesterite devices. The deficient performance of the devices is due to low FF values, which can be attributed to the similar values of the shunt resistance *R*_shunt_ and the series resistance *R*_series_ of the devices. For improved FF values, the *R*_shunt_ values must be significantly higher than the *R*_series_ value. If the values of the device resistance parameters are close, then the degree of squareness of the curve will be affected, leading to low FF values. These can be attributed to the presence of cracks or pin holes during the layer coatings resulting in direct contact of the cathode layer with the back contact.^2^ The contact between the cathode layer and back contact creates an alternative current pathway in the devices. The choice of the device back contact plays a vital role in the overall device performance of kesterite devices. Mo back contact has been used for the best performing kesterite devices. In this study, ITO-coated glass was used as the back contact of these devices, and this choice of back contact can contribute to the low performance of the fabricated devices.^5,9^ CPTSe- and CPTSSe-based devices displayed an improved *V*_oc_ value of ∼0.84 V, while CZTSe and CZTSSe devices displayed a *V*_oc_ value of ∼0.51 V. Table 9 compares some reported selenium-based kesterite devices that were fabricated via the superstrate device architecture with the performance obtained from the fabricated PV devices in this work. PCEs of 1.33 and 3.47% obtained for the CPTSe- and CPTSSe-based PV devices present these novel materials as viable absorber layers for improved kesterite devices. It can be observed that the PCEs for the novel absorber materials contend with already reported devices based on the superstrate device architecture. Also, it is worth noting that the first kesterite PV device had a PCE value of 0.63%,^86^ which was later optimized to yield PCE values >13%.^21^

## 4 Conclusions

In this study, kesterite nanomaterials, CPTSe and CPTSSe, were synthesized via a modified polyol microwave synthesis technique. Notably, the optical, structural, and electrochemical properties of these kesterite compounds were enhanced through the strategic substitution of Zn atoms with Pd atoms. The particle size analysis of the synthesized nanomaterials of CZTSe, CPTSe, CZTSSe, and CPTSSe revealed an increase in particle size for the Pd-containing compounds. This phenomenon was attributed to the larger atomic radius of Pd compared to that of Zn, which led to the enlargement of CPTSe and CPTSSe nanomaterials. The compositional stoichiometry analysis indicated that the CPTSe and CPTSSe nanokesterite materials were shielded from secondary phase formation due to the high energy associated with Cu_Pd_ antisite defect formation. The Pd-kesterite nanomaterials favored ellipsoidal-shaped particles as revealed from DAMMIF ab initio analysis of the SAXS data. CPTSe and CPTSSe nanomaterials exhibited a red-shift of the absorption spectra, reduced band gaps, and the attenuation of defect levels and their concentrations. The incorporation of Pd atoms in the kesterites reduced the activation energy barrier, thereby enhancing the electron mobility and charge-transfer efficiency. Furthermore, the application of CPTSe and CPTSSe nanomaterials as absorber layers in a superstrate solar cell device architecture improved the PCE compared to those of CZTSe- and CZTSSe-based devices. PCE values of 3.47% (CPTSSe) and 1.33% (CPTSe) were achieved, and the devices maintained ∼70% stability after 24 days.

## References

[ref1] Lee T. D.; Ebong A. U. A Review of Thin Film Solar Cell Technologies and Challenges. Renew. Sustain. Energy Rev. 2017, 70, 1286–1297. 10.1016/j.rser.2016.12.028.

[ref2] Nwambaekwe K. C.; John-Denk V.; Douman S. F.; Mathumba P.; Yussuf S. T.; Uhuo O. V.; Ekwere P. I.; Iwuoha E. I. Crystal Engineering and Thin-Film Deposition Strategies towards Improving the Performance of Kesterite Photovoltaic Cell. J. Mater. Res. Technol. 2021, 12, 1252–1287. 10.1016/j.jmrt.2021.03.047.

[ref3] Delbos S. Kësterite Thin Films for Photovoltaics: A Review. EPJ. Photovoltaics 2012, 3, 35004 10.1051/epjpv/2012008.

[ref4] Giraldo S.; Placidi M.; Saucedo E. Kesterite: New Progress toward Earth-Abundant Thin-Film Photovoltaic. In Advanced Micro- and Nanomaterials for Photovoltaics; Elsevier: 2019; pp 93–120.

[ref5] Giraldo S.; Jehl Z.; Placidi M.; Izquierdo-Roca V.; Pérez-Rodríguez A.; Saucedo E. Progress and Perspectives of Thin Film Kesterite Photovoltaic Technology: A Critical Review. Adv. Mater. 2019, 31 (16), 1806692 10.1002/adma.201806692. 30767308

[ref6] Wallace S. K.; Mitzi D. B.; Walsh A. The Steady Rise of Kesterite Solar Cells. ACS Energy Lett. 2017, 2 (4), 776–779. 10.1021/acsenergylett.7b00131.

[ref7] Nazligul A. S.; Wang M.; Choy K. L. Recent Development in Earth-Abundant Kesterite Materials and Their Applications. Sustainability (Switzerland). MDPI AG June 24, 2020; p 12 5138. 10.3390/su12125138.

[ref8] Dale P. J.; Hoenes K.; Scragg J.; Siebentritt S. A Review of the Challenges Facing Kesterite Based Thin Film Solar Cells. In Conf. Rec. IEEE Photovolt. Spec. Conf.; IEEE: 2009; pp 002080–002085.

[ref9] Romanyuk Y. E.; Haass S. G.; Giraldo S.; Placidi M.; Tiwari D.; Fermin D. J.; Hao X.; Xin H.; Schnabel T.; Kauk-Kuusik M.; Pistor P.; Lie S.; Wong L. H. Doping and Alloying of Kesterites. J. Phys. Energy 2019, 1 (4), 044004 10.1088/2515-7655/ab23bc.

[ref10] Guchhait A.; Benny S.; Bhat S. V.; Lawaniya R.; Kumar A.; Dalapati G. K. Cationic Substitution and Doping Approaches for Synthesis of High-Performance Kesterite CZTS(Se) Absorber. In Sulfide and Selenide Based Materials for Emerging Applications: Sustainable Energy Harvesting and Storage Technology; Elsevier: 2022; pp 105–136.

[ref11] Huang D.; Persson C. Band Gap Change Induced by Defect Complexes in Cu_2_ZnSnS_4_. In Thin Solid Films; Elsevier: 2013; Vol. 535, pp 265–269.

[ref12] Dhawale D. S.; Ali A.; Lokhande A. C. Impact of Various Dopant Elements on the Properties of Kesterite Compounds for Solar Cell Applications: A Status Review. Sustain. Energy Fuels 2019, 3 (6), 1365–1383. 10.1039/C9SE00040B.

[ref13] Chen X. Y.; Wang J. L.; Zhou W. H.; Chang Z. X.; Kou D. X.; Zhou Z. J.; Tian Q. W.; Meng Y. N.; Wu S. X. Rational Synthesis of (Cu_1−x_Ag_x_)_2_ZnSnS_4_ Nanocrystals with Low Defect and Tuning Band Gap. Mater. Lett. 2016, 181, 317 10.1016/j.matlet.2016.06.037.

[ref14] Guchhait A.; Su Z.; Tay Y. F.; Shukla S.; Li W.; Leow S. W.; Tan J. M. R.; Lie S.; Gunawan O.; Wong L. H. Enhancement of Open-Circuit Voltage of Solution-Processed Cu_2_ZnSnS_4_ Solar Cells with 7.2% Efficiency by Incorporation of Silver. ACS Energy Lett. 2016, 1 (6), 1256–1261. 10.1021/acsenergylett.6b00509.

[ref15] Giraldo S.; Thersleff T.; Larramona G.; Neuschitzer M.; Pistor P.; Leifer K.; Pérez-Rodríguez A.; Moisan C.; Dennler G.; Saucedo E. Cu_2_ZnSnSe_4_ Solar Cells with 10.6% Efficiency through Innovative Absorber Engineering with Ge Superficial Nanolayer. Prog. Photovoltaics Res. Appl. 2016, 24 (10), 1359–1367. 10.1002/pip.2797.

[ref16] Zhang Q.; Deng H.; Chen L.; Yu L.; Tao J.; Sun L.; Yang P.; Chu J. Cation Substitution Induced Structural Transition, Band Gap Engineering and Grain Growth of Cu_2_Cd_x_Zn_1–x_SnS_4_ Thin Films. J. Alloys Compd. 2017, 695, 482 10.1016/j.jallcom.2016.11.121.

[ref17] Sui Y.; Wu Y.; Zhang Y.; Wang Z.; Wei M.; Yao B. Indium Effect on Structural, Optical and Electrical Properties of Cu_2_In_x_Zn_1–X_SnS_4_ Alloy Thin Films for Solar Cell. Superlattices Microstruct. 2017, 111, 579–590. 10.1016/j.spmi.2017.07.015.

[ref18] Wang X.; Li J.; Zhao Z.; Huang S.; Xie W. Crystal Structure and Electronic Structure of Quaternary Semiconductors Cu_2_ZnTiSe_4_ and Cu_2_ZnTiS_4_ for Solar Cell Absorber. J. Appl. Phys. 2012, 112 (2), 023701 10.1063/1.4736554.

[ref19] Lafond A.; Guillot-Deudon C.; Vidal J.; Paris M.; La C.; Jobic S. Substitution of Li for Cu in Cu_2_ZnSnS_4_: Toward Wide Band Gap Absorbers with Low Cation Disorder for Thin Film Solar Cells. Inorg. Chem. 2017, 56 (5), 2712–2721. 10.1021/acs.inorgchem.6b02865. 28186742

[ref20] Wang W.; Winkler M. T.; Gunawan O.; Gokmen T.; Todorov T. K.; Zhu Y.; Mitzi D. B. Device Characteristics of CZTSSe Thin-Film Solar Cells with 12.6% Efficiency. Adv. Energy Mater. 2014, 4 (7), 1301465 10.1002/aenm.201301465.

[ref21] Saha U.; Biswas A.; Alam M. K. Efficiency Enhancement of CZTSe Solar Cell Using CdS(n)/(Ag_x_Cu_1–x_)_2_ZnSnSe_4_ (p)/Cu_2_ZnSnSe_4_ (p^+^) Structure. Sol. Energy 2021, 221, 314–322. 10.1016/j.solener.2021.04.043.

[ref22] Nwambaekwe K. C.; Masikini M.; Mathumba P.; Ramoroka M. E.; Duoman S.; John-Denk V. S.; Iwuoha E. I. Electronics of Anion Hot Injection-Synthesized Te-Functionalized Kesterite Nanomaterial. Nanomaterials 2021, 11 (3), 794 10.3390/nano11030794. 33808895 PMC8003653

[ref23] Dong H.; Schnabel T.; Ahlswede E.; Feldmann C. Polyol-Mediated Synthesis of Cu_2_ZnSn(S,Se)_4_ Kesterite Nanoparticles and Their Use in Thin-Film Solar Cells. Solid State Sci. 2014, 29, 52–57. 10.1016/j.solidstatesciences.2014.01.006.

[ref24] Huang W. C.; Wei S. Y.; Cai C. H.; Ho W. H.; Lai C. H. The Role of Ag in Aqueous Solution Processed (Ag,Cu)_2_ZnSn(S,Se)_4_ Kesterite Solar Cells: Antisite Defect Elimination and Importance of Na Passivation. J. Mater. Chem. A 2018, 6 (31), 15170–15181. 10.1039/C8TA02950D.

[ref25] Chen S.; Walsh A.; Gong X. G.; Wei S. H. Classification of Lattice Defects in the Kesterite Cu_2_ZnSnS_4_ and Cu_2_ZnSnSe_4_ Earth-Abundant Solar Cell Absorbers. Adv. Mater. 2013, 25 (11), 1522–1539. 10.1002/adma.201203146. 23401176

[ref26] Yuan Z. K.; Chen S.; Xiang H.; Gong X. G.; Walsh A.; Park J. S.; Repins I.; Wei S. H. Engineering Solar Cell Absorbers by Exploring the Band Alignment and Defect Disparity: The Case of Cu- and Ag-Based Kesterite Compounds. Adv. Funct. Mater. 2015, 25 (43), 6733–6743. 10.1002/adfm.201502272.

[ref27] Chakraborty J.; Welzel U.; Mittemeijer E. J. Mechanisms of Interdiffusion in Pd-Cu Thin Film Diffusion Couples. Thin Solid Films 2010, 518 (8), 2010–2020. 10.1016/j.tsf.2009.08.026.

[ref28] Nwambaekwe K. C.; Ramoroka M. E.; Iwuoha E. I. Enhanced Photovoltaic Effects of Microwave-Assisted Polyol-Synthesized Cu_2_(Pd/Zn)SnS_4_ Kesterite Nanoparticles. J. Sci. Adv. Mater. Devices 2023, 8 (2), 100553 10.1016/j.jsamd.2023.100553.

[ref29] Babu G. S. D.; Shajan X. S.; George A.; Parameswaran P.; Murugesan S.; Divakar R.; Mohandas E.; Kumaresan S.; Rao G. M. Low-Cost Hydrothermal Synthesis and Characterization of Pentanary Cu_2_Zn_x_Ni_1–x_SnS_4_ Nanoparticle Inks for Thin Film Solar Cell Applications. Mater. Sci. Semicond. Process. 2017, 63, 127–136. 10.1016/j.mssp.2017.02.015.

[ref30] Cao M.; Shen Y. A Mild Solvothermal Route to Kesterite Quaternary Cu_2_ZnSnS_4_ Nanoparticles. J. Cryst. Growth 2011, 318, 1117–1120. 10.1016/j.jcrysgro.2010.10.071.

[ref31] Jain S.; Singh D.; Vijayan N.; Sharma S. N. Time-Controlled Synthesis Mechanism Analysis of Kesterite-Phased Cu_2_ZnSnS_4_ Nanorods via Colloidal Route. Appl. Nanosci. 2018, 8 (3), 435–446. 10.1007/s13204-018-0781-1.

[ref32] Chen W. C.; Tunuguntla V.; Chiu M. H.; Li L. J.; Shown I.; Lee C. H.; Hwang J. S.; Chen L. C.; Chen K. H. Co-Solvent Effect on Microwave-Assisted Cu_2_ZnSnS_4_ Nanoparticles Synthesis for Thin Film Solar Cell. Sol. Energy Mater. Sol. Cells 2017, 161 (1), 416–423. 10.1016/j.solmat.2016.12.013.

[ref33] Li Z.; Ho J. C. W.; Lee K. K.; Zeng X.; Zhang T.; Wong L. H.; Lam Y. M. Environmentally Friendly Solution Route to Kesterite Cu_2_ZnSn(S,Se)_4_ Thin Films for Solar Cell Applications. RSC Adv. 2014, 4 (51), 26888–26894. 10.1039/C4RA03349C.

[ref34] Nwambaekwe K. C.; Batir V. P.; Dermenji L.; Curmei N. D.; Arushanov E.; Iwuoha E. I. Spray-pyrolyzed Cd-substituted Kesterite Thin-films for Photovoltaic Applications: Post Annealing Conditions and Property Studies. Mater. Chem. Phys. 2023, 301, 127594 10.1016/j.matchemphys.2023.127594.

[ref35] Wu Y.; Zhang Y.; Sui Y.; Wang Z.; Lv S.; Wei M.; Sun Y.; Yao B.; Liu X.; Yang L. Bandgap Engineering of Cu_2_In_x_Zn_1–x_Sn(S,Se)_4_ Alloy Films for Photovoltaic Applications. Ceram. Int. 2018, 44 (2), 1942–1950. 10.1016/j.ceramint.2017.10.137.

[ref36] Pal M.; Mondal O.; Pal M.; Singh R.; Sen D.; Mazumder S. Influence of Doping on Crystal Growth, Structure and Optical Properties of Nanocrystalline CaTiO_3_: A Case Study Using Small-Angle Neutron Scattering. J. Appl. Crystallogr. 2015, 48, 836 10.1107/S1600576715006664.

[ref37] Khawal H. A.; Gawai U. P.; Asokan K.; Dole B. N. Modified Structural, Surface Morphological and Optical Studies of Li^3+^ Swift Heavy Ion Irradiation on Zinc Oxide Nanoparticles. RSC Adv. 2016, 6 (54), 49068–49075. 10.1039/C6RA04803J.

[ref38] Chen S.; Gong X. G.; Walsh A.; Wei S. H. Electronic Structure and Stability of Quaternary Chalcogenide Semiconductors Derived from Cation Cross-Substitution of II-VI and I-III-VI_2_ Compounds. Phys. Rev. B - Condens. Matter Mater. Phys. 2009, 79 (16), 165211 10.1103/PhysRevB.79.165211.

[ref39] Schorr S.; Hoebler H.-J.; Tovar M. A Neutron Diffraction Study of the Stannite-Kesterite Solid Solution Series. Eur. J. Mineral. 2007, 19 (1), 65–73. 10.1127/0935-1221/2007/0019-0065.

[ref40] Schorr S.; Tovar M.; Weber A.; Krauth H.; Honkimaki V.; Schock H. Kesterite–an Alternative Absorber Material for Thin-Film Solar Cells. Acta Crystallogr. Sect. A Found. Crystallogr. 2008, 64 (a1), C59–C60. 10.1107/S0108767308098103.

[ref41] Lambris J. D. Biological Small Angle Scattering: Techniques, Strategies and Tips; Chaudhuri B.; Muñoz I. G.; Qian S.; Urban V. S., Eds.; Advances in Experimental Medicine and Biology; Springer Singapore: Singapore, 2017; 1009; pp 107–129.

[ref42] Trewhella J.; Duff A. P.; Durand D.; Gabel F.; Guss J. M.; Hendrickson W. A.; Hura G. L.; Jacques D. A.; Kirby N. M.; Kwan A. H.; Pérez J.; Pollack L.; Ryan T. M.; Sali A.; Schneidman-Duhovny D.; Schwede T.; Svergun D. I.; Sugiyama M.; Tainer J. A.; Vachette P.; Westbrook J.; Whitten A. E. 2017 Publication Guidelines for Structural Modelling of Small-Angle Scattering Data from Biomolecules in Solution: An Update. Acta Crystallogr. Sect. D Struct. Biol. 2017, 73 (9), 710–728. 10.1107/S2059798317011597. 28876235 PMC5586245

[ref43] Trewhella J.; Jeffries C. M.; Whitten A. E. 2023 Update of Template Tables for Reporting Biomolecular Structural Modelling of Small-Angle Scattering Data. Acta Crystallogr. Sect. D Struct. Biol. 2023, 79 (2), 122–132. 10.1107/S2059798322012141. 36762858 PMC9912924

[ref44] Craievich A. F. Synchrotron SAXS Studies of Nanostructured Materials and Colloidal Solutions: A Review. Mater. Res. 2002, 5 (1), 1–11. 10.1590/S1516-14392002000100002.

[ref45] Sinkó K.; Torma V.; Kovács A. SAXS Investigation of Porous Nanostructures. J. Non. Cryst. Solids 2008, 354 (52–54), 5466–5474. 10.1016/j.jnoncrysol.2008.08.021.

[ref46] Sharma S. K.; Verma D. S.; Khan L. U.; Kumar S.; Khan S. B. Handbook of Materials Characterization; Sharma S. K., Ed.; Springer International Publishing: Cham, 2018.

[ref47] Schnablegger H.; Singh Y. The SAXS Guide. Ant. Paar GmbH: 2013.

[ref48] Shrestha S.; Wang B.; Dutta P. Nanoparticle Processing: Understanding and Controlling Aggregation. Adv. Colloid Interface Sci. 2020, 279, 102162 10.1016/j.cis.2020.102162. 32334131

[ref49] Yang L.; Hu J.; Bai K. Capillary and van Der Waals Force between Microparticles with Different Sizes in Humid Air. J. Adhes. Sci. Technol. 2016, 30 (5), 566–578. 10.1080/01694243.2015.1111834.

[ref50] Dumont M.; Lefebvre W.; Doisneau-Cottignies B.; Deschamps A. Characterisation of the Composition and Volume Fraction of Η′ and η Precipitates in an Al–Zn–Mg Alloy by a Combination of Atom Probe, Small-Angle X-Ray Scattering and Transmission Electron Microscopy. Acta Mater. 2005, 53 (10), 2881–2892. 10.1016/j.actamat.2005.03.004.

[ref51] Szczerba W.; Costo R.; Veintemillas-Verdaguer S.; Morales M. D P.; Thünemann A. F. SAXS Analysis of Single- and Multi-Core Iron Oxide Magnetic Nanoparticles. J. Appl. Crystallogr. 2017, 50 (2), 481–488. 10.1107/S1600576717002370. 28381973 PMC5377343

[ref52] Böer K. W.; Pohl U. W. Band-to-Band Transitions. In Semiconductor Physics; Springer International Publishing: Cham, 2018; pp 455–483.

[ref53] Vogel M. Optical Spectroscopy; Springer. Cham 2024, 126, 315–322. 10.1007/978-3-031-55420-9_20.

[ref54] Adachi S. Properties of Group-IV, III-V and II-VI Semiconductors. Wiley 2005, 10.1002/0470090340.

[ref55] Wang X.; Qarony W.; Cheng P. K.; Ismail M.; Tsang Y. H. Photoluminescence of PdS_2_ and PdSe_2_ Quantum Dots. RSC Adv. 2019, 9 (65), 38077–38084. 10.1039/C9RA07445G. 35541785 PMC9075810

[ref56] Mourdikoudis S.; Pallares R. M.; Thanh N. T. K. Characterization Techniques for Nanoparticles: Comparison and Complementarity upon Studying Nanoparticle Properties. Nanoscale 2018, 10 (27), 12871–12934. 10.1039/C8NR02278J. 29926865

[ref57] Ravindra N. M.; Ganapathy P.; Choi J. Energy Gap-Refractive Index Relations in Semiconductors - An Overview. Infrared Phys. Technol. 2007, 50 (1), 21–29. 10.1016/j.infrared.2006.04.001.

[ref58] Dubey R. S.; Saravanan S.; Kalainathan S. Performance Enhancement of Thin Film Silicon Solar Cells Based on Distributed Bragg Reflector & Diffraction Grating. AIP Adv. 2014, 10.1063/1.4904218. PMC4494017 26088994

[ref59] Yan J.; Liu X.; Ma C.; Huang Y.; Yang G. All-Dielectric Materials and Related Nanophotonic Applications. Mater. Sci. Eng. R Reports 2020, 141, 100563 10.1016/j.mser.2020.100563.

[ref60] Brebels J.; Manca J. V.; Lutsen L.; Vanderzande D.; Maes W. High Dielectric Constant Conjugated Materials for Organic Photovoltaics. J. Mater. Chem. A 2017, 5 (46), 24037–24050. 10.1039/C7TA06808E.

[ref61] von Hippel A. R.; Morgan S. O. Dielectric Materials and Applications. J. Electrochem. Soc. 1955, 102 (3), 68C 10.1149/1.2430014.

[ref62] Li Z.; Peng B.; Lin M. L.; Leng Y. C.; Zhang B.; Pang C.; Tan P. H.; Monserrat B.; Chen F. Phonon-Assisted Electronic States Modulation of Few-Layer PdSe_2_ at Terahertz Frequencies. *npj 2D Mater*. Appl. 2021, 5 (1), 87 10.1038/s41699-021-00268-3.

[ref63] Sava F.; Diagne O.; Galca A. C.; Simandan I. D.; Matei E.; Burdusel M.; Becherescu N.; Becherescu V.; Mihai C.; Velea A. Secondary Crystalline Phases Influence on Optical Properties in Off-Stoichiometric Cu_2_S–ZnS–SnS_2_ Thin Films. Materials (Basel). 2020, 13, 4624 10.3390/ma13204624. 33081362 PMC7603050

[ref64] Soltanmohammadi M.; Spurio E.; Gloystein A.; Luches P.; Nilius N. Photoluminescence Spectroscopy of Cuprous Oxide: Bulk Crystal versus Crystalline Films. Phys. status solidi 2023, 220 (9), 2200887 10.1002/pssa.202200887.

[ref65] Liu Y.; Kong D.-Y.; You H.; Chen C.; Lin X.; Brugger J. Structural and Optical Properties of the Cu_2_ZnSnSe_4_ Thin Films Grown by Nano-Ink Coating and Selenization. J. Mater. Sci. Mater. Electron. 2013, 24 (2), 529–535. 10.1007/s10854-012-0970-8.

[ref66] Patel P.; Solanki R. G.; Gupta P.; Sujata K. M.; Balachandran B. Photoluminescence Properties of Copper Selenide Nanoparticles for Red LEDs and Lasers. MRS Adv. 2023, 8 (17), 960–968. 10.1557/s43580-023-00547-9.

[ref67] Nwambaekwe K. C. Tellurium Attenuation of Kesterite Band Gap for Improved Photovoltaic Efficiency; University of the Western Cape: Bellville, South Africa, 2018. https://etd.uwc.ac.za/xmlui/bitstream/handle/11394/6656/nwambaekwe_nsc_2019_36.pdf?sequence=1&isAllowed=y.

[ref68] Eskalen H.; Çeşme M. Carbon Dots from Turnip Juice: Synthesis, Characterization and Investigation of PH-Dependent Optical Properties. Bilecik Şeyh Edebali Üniversitesi Fen Bilim. Derg. 2021, 8 (2), 924–930. 10.35193/bseufbd.979306.

[ref69] Jadreško D.; Zelić M. Cyclic Multipulse Voltammetric Techniques. Part I: Kinetics of Electrode Processes. J. Electroanal. Chem. 2013, 707, 20–30. 10.1016/j.jelechem.2013.08.011.

[ref70] Ameur Z. O.; Husein M. M. Electrochemical Behavior of Potassium Ferricyanide in Aqueous and (w/o) Microemulsion Systems in the Presence of Dispersed Nickel Nanoparticles. Sep. Sci. Technol. 2013, 48 (5), 681–689. 10.1080/01496395.2012.712594.

[ref71] Popescu A. M.; Constantin V.; Cojocaru A.; Olteanu M. Electrochemical Behaviour of Copper (II) Chloride in Choline Chloride-Urea Deep Eutectic Solvent. Rev. Chim. 2011, 62 (2), 206–211.

[ref72] Budnikova Y. H.; Dudkina Y. B.; Khrizanforov M. N. Redox-Induced Aromatic C-H Bond Functionalization in Metal Complex Catalysis from the Electrochemical Point of View. In Inorganics; Multidisciplinary Digital Publishing Institute: 2017; p 70.

[ref73] Yuan X.; Xue S.; Liao J.; Peng F.; Shao L.; Zhang J. A Robust Approach to Fabricate CZTSSe Absorber Layer for Solar Cells via a Self-Selenizations Process Conducted by Concentrated Selenium Solution. Mater. Res. Express 2018, 5 (1), 016413 10.1088/2053-1591/aaa6df.

[ref74] Rondiya S.; Wadnerkar N.; Jadhav Y.; Jadkar S.; Haram S.; Kabir M. Structural, Electronic, and Optical Properties of Cu_2_NiSnS_4_: A Combined Experimental and Theoretical Study toward Photovoltaic Applications. Chem. Mater. 2017, 29 (7), 3133–3142. 10.1021/acs.chemmater.7b00149.

[ref75] Ray A.; Roy A.; Sadhukhan P.; Chowdhury S. R.; Maji P.; Bhattachrya S. K.; Das S. Electrochemical Properties of TiO_2_-V_2_O_5_ Nanocomposites as a High Performance Supercapacitors Electrode Material. Appl. Surf. Sci. 2018, 443, 581–591. 10.1016/j.apsusc.2018.02.277.

[ref76] Cao C.; Vernon R. E.; Schwarz W. H. E.; Li J. Understanding Periodic and Non-Periodic Chemistry in Periodic Tables. In Frontiers in Chemistry; Frontiers Media S.A.: 2021; p 813. 10.3389/fchem.2020.00813 PMC7818537 33490030

[ref77] Ganesh V.; Pal S. K.; Kumar S.; Lakshminarayanan V. Self-Assembled Monolayers (SAMs) of Alkoxycyanobiphenyl Thiols on Gold - A Study of Electron Transfer Reaction Using Cyclic Voltammetry and Electrochemical Impedance Spectroscopy. J. Colloid Interface Sci. 2006, 296 (1), 195–203. 10.1016/j.jcis.2005.08.051. 16209874

[ref78] Vasilescu C.; Drob S. I.; Osiceanu P.; Calderon Moreno J. M.; Prodana M.; Ionita D.; Vasilescu E.; Popa M. Behavior of Passive Film on New Ti-Zr-Ta-Ag Alloy Surface in Simulated Biofluid. ECS Meet. Abstr. 2016, MA2016-01 (15), 954 10.1149/MA2016-01/15/954.

[ref79] Vasilescu C.; Drob S. I.; Osiceanu P.; Moreno J. M. C.; Prodana M.; Ionita D.; Demetrescu I.; Marcu M.; Popovici I. A.; Vasilescu E. Microstructure, Surface Characterization, and Electrochemical Behavior of New Ti-Zr-Ta-Ag Alloy in Simulated Human Electrolyte. Metall. Mater. Trans. A Phys. Metall. Mater. Sci. 2017, 48 (1), 513–523. 10.1007/s11661-016-3774-2.

[ref80] Dang H. L. T.; Dao V. D.; Vu N. H.; Quang D. V.; Vu H. H. T.; Nguyen T. H.; Mohamed I. M. A.; Hoang X. C.; Vu D. A.; Tuan P. A. Balance between the Explored Pt Counter Electrode in an Electrolyte Medium and the Photoanode for Highly Efficient Liquid-Junction Photovoltaic Devices. J. Sci. Adv. Mater. Devices 2020, 5 (2), 180–184. 10.1016/j.jsamd.2020.03.002.

[ref81] Jeong H.; Pak Y.; Hwang Y.; Song H.; Lee K. H.; Ko H. C.; Jung G. Y. Enhancing the Charge Transfer of the Counter Electrode in Dye-Sensitized Solar Cells Using Periodically Aligned Platinum Nanocups. Small 2012, 8 (24), 3757–3761. 10.1002/smll.201201214. 22972565

[ref82] Franckevičius M.; Pakštas V.; Grincienė G.; Kamarauskas E.; Giraitis R.; Nekrasovas J.; Selskis A.; Juškėnas R.; Niaura G. Efficiency Improvement of Superstrate CZTSSe Solar Cells Processed by Spray Pyrolysis Approach. Sol. Energy 2019, 185, 283–289. 10.1016/j.solener.2019.04.072.

[ref83] Terlemezoglu M.; Sürücü B.; Dogru C.; Güllü H. H.; Ciftpinar E. H.; Erçelebi; Parlak M. CZTSSe Thin Films Fabricated by Single Step Deposition for Superstrate Solar Cell Applications. J. Mater. Sci. Mater. Electron. 2019, 30 (12), 11301–11306. 10.1007/s10854-019-01477-9.

[ref84] Wang C. L.; Manthiram A. Low-Cost CZTSSe Solar Cells Fabricated with Low Band Gap CZTSe Nanocrystals, Environmentally Friendly Binder, and Nonvacuum Processes. ACS Sustain. Chem. Eng. 2014, 2 (4), 561–568. 10.1021/sc400465m.

[ref85] Zhang Y.; Sun Y.; Wang H.; Yan H. A Facile Non-Vacuum-Based Cu_2_ZnSnSe_4_ Superstrate Solar Cell with 2.44% Device Efficiency. Phys. Status Solidi Appl. Mater. Sci. 2016, 213 (5), 1324–1328. 10.1002/pssa.201532648.

[ref86] Katagiri H.; Sasaguchi N.; Hando S.; Hoshino S.; Ohashi J.; Yokota T. Preparation and Evaluation of Cu_2_ZnSnS_4_ Thin Films by Sulfurization of E-B Evaporated Precursors. Sol. Energy Mater. Sol. Cells 1997, 49 (1–4), 407–414. 10.1016/S0927-0248(97)00119-0.

